# Supplementary material for: United Kingdom Research study into Ethnicity And COVID-19 outcomes in Healthcare workers (UK-REACH): a retrospective cohort study using linked routinely collected data, study protocol
Source: BMJ Open. 2021 Jun 28;11(6):e046392. doi: 10.1136/bmjopen-2020-046392 (PMC8245289; doi:10.1136/bmjopen-2020-046392)
Supplement: Author's manuscript [file bmjopen-2020-046392.draft_revisions.pdf]

BMJ Open is committed to open peer review. As part of this commitment we make the peer review history of every article we publish publicly available.

When an article is published we post the peer reviewers' comments and the authors' responses online. We also post the versions of the paper that were used during peer review. These are the versions that the peer review comments apply to.

The versions of the paper that follow are the versions that were submitted during the peer review process. They are not the versions of record or the final published versions. They should not be cited or distributed as the published version of this manuscript.

BMJ Open is an open access journal and the full, final, typeset and author-corrected version of record of the manuscript is available on our site with no access controls, subscription charges or pay-per-view fees (http://bmjopen.bmj.com).

If you have any questions on BMJ Open's open peer review process please email info.bmjopen@bmj.com

BMJ Open

United Kingdom Research study into Ethnicity And COVID-19 outcomes in Healthcare workers (UK-REACH): a retrospective cohort study using linked routinely collected data, study protocol

Journal:	*BMJ Open*
Manuscript ID	bmjopen-2020-046392
Article Type:	Protocol
Date Submitted by the Author:	12-Nov-2020
Complete List of Authors:	Teece, Lucy; University of Leicester, Biostatistics Research Group, Department of Health Sciences<br>Gray, Laura; University of Leicester, Biostatistics Research Group, Department of Health Sciences<br>Melbourne, Carl; University of Leicester, Genetic Epidemiology Research Group, Department of Health Sciences<br>Orton, Chris; Swansea University Medical School, Population Data Science<br>Ford, David; Swansea University Medical School, Population Data Science<br>Martin, Christopher; University of Leicester, Department of Respiratory Sciences; University Hospitals of Leicester NHS Trust, Department of Infection and HIV Medicine<br>McAllister, David; University of Glasgow, Institute of Health and Wellbeing<br>Khunti, Kamlesh; Leicester General Hospital, Diabetes Research Centre, University of Leicester; Leicester General Hospital, Leicester Real World Evidence Unit, University of Leicester<br>Tobin, Martin; University of Leicester, Genetic Epidemiology Research Group, Department of Health Sciences; NIHR Leicester Biomedical Research Centre Respiratory Diseases, Glenfield Hospital<br>John, Catherine; University of Leicester, Genetic Epidemiology Research Group, Department of Health Sciences<br>Guyatt, Anna; University of Leicester, Genetic Epidemiology Research Group, Department of Health Sciences<br>Abrams, Keith; University of Leicester, Biostatistics Research Group, Department of Health Sciences<br>Pareek, Manish; University of Leicester, Department of Respiratory Sciences; University Hospitals of Leicester NHS Trust, Department of Infection and HIV Medicine
Keywords:	COVID-19, EPIDEMIOLOGY, Public health < INFECTIOUS DISEASES, Adult intensive & critical care < INTENSIVE & CRITICAL CARE

For peer review only - http://bmjopen.bmj.com/site/about/guidelines.xhtml

I, the Submitting Author has the right to grant and does grant on behalf of all authors of the Work (as defined in the below author licence), an exclusive licence and/or a non-exclusive licence for contributions from authors who are: i) UK Crown employees; ii) where BMJ has agreed a CC-BY licence shall apply, and/or iii) in accordance with the terms applicable for US Federal Government officers or employees acting as part of their official duties; on a worldwide, perpetual, irrevocable, royalty-free basis to BMJ Publishing Group Ltd ("BMJ") its licensees and where the relevant Journal is co-owned by BMJ to the co-owners of the Journal, to publish the Work in this journal and any other BMJ products and to exploit all rights, as set out in our licence.

The Submitting Author accepts and understands that any supply made under these terms is made by BMJ to the Submitting Author unless you are acting as an employee on behalf of your employer or a postgraduate student of an affiliated institution which is paying any applicable article publishing charge ("APC") for Open Access articles. Where the Submitting Author wishes to make the Work available on an Open Access basis (and intends to pay the relevant APC), the terms of reuse of such Open Access shall be governed by a Creative Commons licence – details of these licences and which Creative Commons licence will apply to this Work are set out in our licence referred to above.

Other than as permitted in any relevant BMJ Author's Self Archiving Policies, I confirm this Work has not been accepted for publication elsewhere, is not being considered for publication elsewhere and does not duplicate material already published. I confirm all authors consent to publication of this Work and authorise the granting of this licence.

For peer review only

United Kingdom Research study into Ethnicity And COVID-19 outcomes in Healthcare workers (UK-REACH): a retrospective cohort study using linked routinely collected data, study protocol

Lucy Teece[1], Laura J Gray[1], Carl Melbourne[2], Christopher Orton[3], David V Ford[3], Christopher A Martin[4,5], David A McAllister[6], Kamlesh Khunti[7,8], Martin Tobin[2,9], Catherine John[2], Anna Guyatt[2], Keith R Abrams[1], Manish Pareek[4,5]

For the UK-REACH Study Team.

[1]Biostatistics Research Group, Department of Health Sciences, University of Leicester, Leicester, UK.

[2]Genetic Epidemiology Research Group, Department of Health Sciences, University of Leicester, Leicester, UK.

[3]Population Data Science, Swansea University Medical School, Swansea University, Swansea, UK.

[4]Department of Respiratory Sciences, University of Leicester, Leicester, UK.

[5]Department of Infection and HIV Medicine, University Hospitals of Leicester NHS Trust, Leicester, UK.

[6]Institute of Health and Wellbeing, University of Glasgow, Glasgow, UK.

[7]Diabetes Research Centre, University of Leicester, Leicester General Hospital, Leicester, UK.

[8]Leicester Real World Evidence Unit, University of Leicester, Leicester General Hospital, Leicester, UK.

[9]National Institute for Health Research, Leicester Respiratory Biomedical Research Centre, Glenfield Hospital, Leicester, UK

Key words: COVID-19, diagnosis, hospitalisation, mortality, healthcare workers, ethnicity.

Corresponding author (& contact info): Manish Pareek (manish.pareek@leicester.ac.uk)

Word count main text: 4,677

Word count abstract: 220

Target Journals: BMJ Open (will consider publishing without peer review protocols that have formal ethical approval and funding from a recognised, open access advocating research-funding body).

Date: 01 September 2020

For peer review only

ABSTRACT

Introduction

Coronavirus disease 2019 (COVID-19) has spread rapidly worldwide, causing significant morbidity and mortality. People from ethnic minorities, particularly those working in healthcare settings, have been disproportionately affected. Current evidence of the association between ethnicity and COVID-19 outcomes in people working in healthcare settings is insufficient to inform plans to address health inequalities.

Methods and analysis

This study combines anonymised human resource databases with professional registration and NHS datasets to assess associations between ethnicity and COVID-19 diagnosis, hospitalisation, and death in healthcare workers in the UK. Adverse COVID-19 outcomes will be assessed between 01-02-2020 (date following first confirmed COVID-19 case in UK) and date of data extraction (30-09-20 at a minimum, allowing 8 months follow-up).

Planned analyses include multivariable Poisson, logistic, and flexible parametric time-to-event regression within each country, adjusting for core predictors, followed by meta-analysis to produce combined effect estimates for the UK. Mediation analysis methods will be explored to examine associations between ethnicity and COVID-19 outcomes.

Ethics and dissemination

Ethical approval for the UK-REACH programme has been obtained via the expedited HRA COVID-19 processes (REC ref: 20/HRA/4718, IRAS ID: 288316). Research information will be anonymised via the Secure Anonymised Information Linkage (SAIL) Databank before release to researchers. Study results will be submitted for publication in an open access peer-reviewed journal and made available on our dedicated website (https://uk-reach.org/).

For peer review only

For peer review only - http://bmjopen.bmj.com/site/about/guidelines.xhtml

ARTICLE SUMMARY

Strengths and limitations of this study

1. First large UK-wide study assessing the associations between ethnicity and COVID-19 outcomes in people who work in healthcare settings.
2. Ambitious linkage of multiple anonymised human resource and professional registration databases with NHS datasets, aiming to provide coverage of the whole of the UK.
3. Broad and comprehensive analysis that aims to unpick the associations between ethnicity, occupational group, and multiple COVID-19 outcomes while adjusting for core predictors.
4. Access to highly detailed human resource databases (including Electronic Staff Record for England and Wales) enables granular analyses of ethnicity, occupation, and COVID-19 exposures.
5. A key limitation is the lack of a single UK-wide database capturing those actively working within healthcare settings, which may reduce our ability to identify all health care workers across all regions. However, we are making every effort to identify as many health care workers as possible, including contacting organisations to identify non-clinical ancillary staff (e.g. cleaners, caterers, porters) and those working in non-NHS settings.

For peer review only

INTRODUCTION

Coronavirus disease 2019 (COVID-19) has been declared a global pandemic. Since the first reported cases in December 2019, over 34.8 million cases and 1 million deaths have been reported worldwide [1]. Consistent evidence identifies advanced age, male sex, socio-economic deprivation, and chronic comorbidities as core predictors for COVID-19 adverse outcomes [2-5].

The COVID-19 pandemic has disproportionately impacted historically disadvantaged populations [6]. In the UK, higher infection and mortality rates are observed in areas with greater deprivation [7] and amongst ethnic minorities [8-12]. This has prompted calls to prioritise the publication of data disaggregated by ethnicity and to conduct research which increases our understanding of risk in these populations [13, 14]. Ethnicity is a complex construct which represents social, biological, or genetic differences between populations [15]. Potential explanations for COVID-19 related health disparities between ethnic groups include socioeconomic, cultural, behavioural, biological, or pathophysiological differences [16]. It is recommended that investigations into ethnicity-related health inequality should also consider interrelated factors such as deprivation, religion, and pre-existing conditions. This approach serves to highlight the interplay between ethnic disparities and economic inequality [13] and disentangle the independent importance of these factors [14].

Those working in healthcare settings, hereafter referred to as Health Care Workers (HCWs), have a greater risk of severe acute respiratory syndrome coronavirus 2 (SARS-CoV-2) exposure and transmission due to the number of COVID-19 cases requiring medical intervention [17]. A Scottish linkage study concluded over a sixth of hospitalised COVID-19 cases were HCWs or members of their households [18]. There is concern that HCWs from ethnic minority groups are at increased risk of adverse outcomes from COVID-19. However, the interplay between ethnicity and healthcare occupation and its impact on COVID-19 outcomes is unclear. Publicly available data indicates that individuals from ethnic minorities account for 65-76% of deaths reported in clinical HCWs, despite contributing less than 20% of the NHS workforce [19, 20]. We must understand differences in risks in these populations to protect the most vulnerable HCWs and maintain a functioning healthcare system [18]. However, the quality of data available to evaluate COVID-19 outcomes in HCWs, and the impact of ethnicity on these outcomes, remains poor and limited by small sample size.

The United Kingdom Research study into Ethnicity And COVID-19 diagnosis and outcomes in Healthcare workers (UK-REACH) programme will rapidly examine differences in COVID-19 diagnosis, clinical outcomes, professional practices, and well-being among HCWs from different ethnicities. It will provide rapid evidence through five interlinked work packages; a large data linkage cohort study, a longitudinal cohort study, a legal/ethical analysis, a qualitative work stream, and the development of a stakeholder working group. Here we describe the protocol for the large data linkage cohort study, which will bring together anonymised human resource, professional registration, and NHS datasets within a Trusted Research Environment [21]. This linked database will be used to assess the relationship between ethnicity and COVID-19 diagnosis, hospitalisation, and death in HCWs.

Objectives

Our primary aim is to determine whether COVID-19 diagnosis, hospitalisation, and mortality rates differ between ethnic and occupational groups in HCWs. We will conduct both a broad analysis,

For peer review only - http://bmjopen.bmj.com/site/about/guidelines.xhtml

encompassing all those registered as HCWs on 01-02-2020 in the UK, and a detailed sub-study analysis in those actively working during the COVID-19 pandemic, incorporating more granular ethnicity, occupation, and exposure information.

Considering current evidence, we hypothesise that HCWs from ethnic minority backgrounds will have increased risk of COVID-19 diagnosis and adverse outcomes compared to their White counterparts regardless of occupation.

For peer review only

For peer review only - http://bmjopen.bmj.com/site/about/guidelines.xhtml

METHODS

Study design

We will utilise data from multiple linked electronic health record (EHR) databases to investigate the study hypotheses within a cohort of HCWs. The cohort study will begin on 01-02-2020 (date following first confirmed COVID-19 case in UK) and follow participants for as long as possible (i.e. to date of data extraction: 30-09-20 at a minimum allowing 8 months follow-up).

Data sources

Our primary data sources are the NHS human resource (HR) and payroll databases, namely the Electronic Staff Record (ESR), capturing approximately 1.4 million staff paid through the NHS in England and Wales, and the Scottish Workforce Information Standard System (SWISS) including approximately 170,000 staff directly employed by NHS Scotland. As well as clinical staff, these databases include NHS paid ancillary and administrative services staff, and those in management roles. These HR databases cover NHS organisations including hospital trusts, ambulance services, and clinical commissioning groups with comprehensive employment data during the cohort period of interest (Table 1). We also aim to access HR data from non-NHS ancillary worker contractor companies to capture non-clinical staff and those working in facilities management and care coordination positions.

To enable wider coverage of the UK and capture workers in non-NHS sectors (e.g. community and primary care), the HR data will be supplemented by health and social care regulators, where available. These include the General Medical Council (GMC), Nursing and Midwifery Council (NMC), General Dental Council (GDC), General Pharmaceutical Council (GPhC), General Optical Council (GOC), Pharmaceutical Society Northern Ireland (PSNI), and Health and Care Professions Council (HCPC). Where possible, we will obtain data for all HCWs registered to these regulators on the cohort inception date (01-02-2020). As these registries provide less information on specific occupational role and workplace location, it's not possible to identify those actively working during the study period. All included bodies provide UK-wide coverage, except pharmaceutical bodies GPhC and PSNI, which regulate Great Britain and Northern Ireland separately. Table 1 compares the coverage, inclusion, and data available from each registration database.

Given the differences in coverage and data across databases, the analysis will be two-fold. Firstly, a broad analysis will address the main objective using the full UK-wide cohort for any HCW employed or registered on 01-02-2020. Secondly, a detailed HR sub-study will incorporate additional information on ethnicity, occupational role, and potential for SARS-CoV-2 exposure (including whether an individual actively worked during the study period), and changes to these throughout the study period.

COVID-19 diagnosis, hospitalisation, and mortality data will be gathered from several routinely collected EHR databases. We aim to obtain primary and secondary care datasets as well as intensive care audit data (such as ICNARC for England and Wales) and mortality (Office for National Statistics, ONS) for each country. We also plan to incorporate specialised COVID-19 related databases, including COVID-19 surveillance data (COVID-19 Hospitalisation in England Surveillance System, CHESS), COVID Symptom Study app data [22], and Public Health England (PHE) Pillar 1 (within hospital) and Pillar 2

For peer review only - http://bmjopen.bmj.com/site/about/guidelines.xhtml

(community-based) testing data, where available. Additional datasets, such as PHE Pillar 3 and 4, will be sought and incorporated if obtained.

It may not be possible to access all outcome datasets for England, Wales, Scotland, and Northern Ireland. Therefore, outcomes will be assessed separately in each country and results combined to produce UK-wide estimates.

Data linkage

Data will be merged, linked, anonymised, and stored at the Secure Anonymised Information Linkage (SAIL) databank [21] (Figure 1). SAIL works in partnership with a trusted third party, the NHS Wales Informatics Service (NWIS), to provide these services and ensure processing of personal data is kept separated from the research environment.

The process for anonymization and linkage is as follows: Information from the HR and regulator databases will be separated into identifiable data and other administrative data. The identifiable data will be sent directly to NWIS and anonymised by encrypting identifiers into a single anonymous ID. Databases will be linked using this ID to identify the study cohort and de-duplicate individuals present in more than one of the HR and regulator databases. Other administrative data will be sent directly to SAIL, who will use the linkage key (subsequently removed to prohibit reverse engineering of re-identification) to match to the anonymised data. NWIS will share the relevant identifiers from the study cohort with health record providers, such as NHS Digital, NHS Scotland, and the Northern Ireland Honest Broker Service, to establish linkage. Extracts of relevant health records will be sent to SAIL following the same split-file process. Identifiable health record data will be kept entirely within the NHS, with linked anonymised records matched in SAIL for the project analysis to take place. Once the linkage process is complete, the identifiable data will be deleted in accordance with the contractual arrangement between NWIS and SAIL.

We will develop a process to enable access to the UK-REACH data for other researchers once this study is complete. This process will engage with the above health record providers to establish onward sharing in partnership with the SAIL Databank.

Study population

The cohort will include all adult (aged 16 years and over) HCWs captured by HR records or registered with at least one professional regulator on 01-02-2020, excluding those which linkage to EHRs is unsuccessful. HCWs with missing ethnicity, occupation, age, sex, or postcode will be excluded from the main analysis. For the HR sub-study, only those actively working will be included, thus excluding those on parental, sickness, or disability leave for the duration of study period.

Observation period

Follow-up commences on the index date (01-02-2020) (or date of return/commencement of employment for the HR sub-study) and continue until either their death date (COVID-19 related or due to other causes) or the study end date (30-09-20 at the earliest). For the HR sub-study, those leaving NHS employment during the study period will be included up until this date, at which point they will be censored.

For peer review only - http://bmjopen.bmj.com/site/about/guidelines.xhtml

Main exposures

The main exposure of interest is ethnicity, which is likely to be inconsistently recorded across databases. We will primarily assign ethnicity using staff HR records (ESR or SWISS) and registration databases (if not included/missing from HR records). If ethnicity is missing from these databases, linked EHRs will be assessed. Those with ethnicity recorded as 'not stated' or with completely missing ethnicity information will be excluded from the main analyses. Ethnicity will be categorised based on ONS groupings (Table 2).

A more granular analysis will incorporate subdivisions of ethnicity (particularly within Asian/Asian British category) if a sufficient number of events are observed.

Occupational group (and its interaction with ethnicity) is also of primary interest. We will assign occupation for the main cohort using data recorded on 01-02-2020 from either HR records or data held by the regulating bodies and the following categories informed by version 17.1 of the NHS Occupational Code Manual (*NHS Digital*):

- Ambulance staff
- Administrations and estates staff
- Health care assistants and other support staff
- Medical staff
- Dental staff
- Nursing, midwifery and health visiting staff
- Nursing, midwifery and health visiting learners
- Scientific, therapeutic and technical staff
- Healthcare sciences
- General payments

A more granular analysis will incorporate subdivisions of occupational groups (particularly the Medical staff group) if a sufficient number of events are observed.

Outcomes

The main outcomes are COVID-19 diagnosis (confirmed and suspected, confirmed only), hospitalisation (all, COVID-19 specific, ITU admission) and mortality (all-cause, COVID-19 specific) (Table 3 : COVID-19 outcomes and definitions, identified via listed data sources, to be asessed in UK healthcare worker cohort and within listed subgroups, via listed analysis approach in the planned study).

Predictors

We will investigate and adjust for the following core predictors: age, sex, comorbidities, and socio-economic deprivation (Table 4).

Elevated body mass index (BMI), obesity, and smoking are also considered key predictors for adverse COVID-19 outcomes [9, 23, 24] however are likely to be missing for large proportions of the identified cohort. Furthermore, missingness in EHR data is unlikely to be random, preventing standard imputation approaches. Thus, BMI and smoking will not be included as independent predictors in the

For peer review only - http://bmjopen.bmj.com/site/about/guidelines.xhtml

primary study but instead in a sensitivity analysis exploring COVID-19 outcomes within HCWs where this information is available in primary or secondary care medical records, within 24 months prior to the study start date.

HR sub-study

This sub-study will consider only those undertaking active employment in the NHS during the study observation period and will be conducted only in regions where data are available.

Additional ethnicity related predictors include religious belief, nationality, immigration status, and country of birth. Additional occupation predictors include primary area of work, role description, full/part time, patient/non-patient facing [18], time in current role, organisation type, salary grade, length of service, and seniority. Additional exposure related predictors include absence information and total hours worked. Changes in these predictors will be captured throughout the study period. Time varying covariates will be incorporated to enable the assessment of the impact of changes in COVID-19 exposure over time.

Patient and Public Involvement

We have been working with organisations that regulate and represent ethnic minority HCWs, including the GMC, NMC, Royal Colleges, and professional associations such as the British Association of Physicians of Indian Origin. They were involved in identifying research questions and deciding study methodology, and are included as members of the study delivery team or collaborators. These organisations are part of the stakeholder group, meeting monthly to monitor study progress and outputs, provide advice on study delivery, and disseminate research findings. Representatives from ethnic minority healthcare professionals, including those who have contracted COVID-19, will also input into this stakeholder group. A Professional Expert Panel (PEP) of doctors, nurses, and ancillary staff from varying ethnic backgrounds and genders, will meet bi-monthly to provide advice and lived experience related to certain aspects of the project.

For peer review only

STATISTICAL ANALYSIS PLAN

Statistical principles

Reporting will be in line with STROBE [25] and RECORD [26] guidelines. 95% confidence intervals will be reported throughout and p-values<0.05 will indicate statistical significance. Multiple testing adjustments will not be made but emphasis will be on effect size rather than statistical significance. Deviations to this protocol and analysis plan will be outlined with justification in the final reporting of the results.

Sample size

Sample size will be driven by the number of HCWs captured by the registration databases, with complete exposure information and successful linkage to healthcare data. The analysis plan is informed by likely event rates for each outcome, undertaking more sophisticated analyses for outcomes with sufficient power to provide meaningful results.

We will consider our largest primary data source (ESR) which captures 1.4 million NHS employees in England and Wales, for a conservative estimate of potential precision. The COVID-19 Infection Survey estimate 6.2% (95% CI 5.0% to 7.6%) of adults in England would be seropositive for anti-SARS-CoV-2 antibodies [27]. Broadly assuming an equal infection rate in Wales, and that HCWs captured by the ESR represent the general population with a similar infection rate, we could expect to capture approximately 86,600 HCWs with history of COVID-19. A large proportion of the infected population will be asymptomatic, and a recent US study projected that 63% of infected cases went undiagnosed [28]. Assuming a similar diagnosis rate in England and Wales, we could expect to identify 32,042 COVID-19 cases. Of adults diagnosed with SARS-CoV-2 infections the estimated hospitalisation rate is 25% [29] and mortality rate is 6.4% [28]. We could therefore expect to observe a minimum of 8,000 hospitalisations and 2,050 deaths resulting from COVID-19.

NHS workforce statistics data show 79.2% of the NHS workforce is White, 10.0% Asian, and 6.1% Black. If associations between ethnicity and COVID-19 outcomes represent those observed in the general population, we could report differences between COVID-19 infection for Asian and Black ethnic categories compared to White with 0.1% and 0.2% precision respectively. Similarly, in those with confirmed SARS-CoV-2 infection we could report differences in COVID-19 mortality between Asian and Black ethnic categories, compared to White, with 0.9% and 0.8% precision respectively.

We will supplement the ESR with other HR databases and professional registration datasets, including SWISS, with an additional 170,000 HCWs in Scotland, and the GMC, with an additional 336,000 GPs not captured by ESR. This increases the total number of HCWs incorporated into our analyses and should ensure sufficient numbers of outcome events.

Missing data

Missing information for ethnicity, occupation, and other predictor variables will be reported and missing data patterns investigated. To improve completeness, we will utilise information from multiple data sources. Those with completely missing ethnicity information will be excluded from the

For peer review only - http://bmjopen.bmj.com/site/about/guidelines.xhtml

main analysis. Comorbidity information will be identified using primary care records; thus, absence of comorbidity codes will be interpreted as absence of comorbidity (i.e. not missing).

We will report the coverage of each analysis and the number (%) of HCWs included. Sensitivity to the exclusion of missing data will be assessed using multiple imputation methods.

Baseline characteristics

The frequency (%) of HCWs by ethnicity and occupational group will be reported. Core predictors (age, sex, comorbidity, and deprivation) will be summarised by ethnicity and occupational group using frequency (%) for categorical measures and mean (standard deviation) or median (interquartile range) for continuous. We will compare core predictors between those with recorded and missing ethnicity information.

For the HR sub-study, additional ethnicity, occupation, and exposure predictors will be summarised alongside the core predictors where available. Changes in roles, exposure, and absences over time will be described across ethnicity category and occupational group.

All baseline data will be presented for the whole cohort and by country.

A flowchart, using the STROBE format, will present cohort selection for both the broad and HR sub-study analyses, with exclusions explained. Average and overall follow-up time will be summarised for each cohort, by ethnicity, occupational group, and country.

Outcome incidence

Adjusted incidence rates for each outcome will be calculated for each country where available. Meta-analysis methods will give an overall UK incidence rate estimate. Adjusted incidence rates by ethnicity and occupation will be calculated in the same way. All incidence rates will be adjusted for the four core predictors. The incidence of each outcome will be reported as the rate of cases in the final UK HCW cohort. Incidence rate will additionally be reported within subgroups who have experienced preceding COVID-19 events (Table 3 : COVID-19 outcomes and definitions, identified via listed data sources, to be asessed in UK healthcare worker cohort and within listed subgroups, via listed analysis approach in the planned study), the time since diagnosis will be summarised and cumulative incidence curves will be plotted.

Analysis approach

The relationship between ethnicity and COVID-19 outcomes will be assessed using Poisson, logistic, and flexible parametric time-to-event regression models (Table 3 : COVID-19 outcomes and definitions, identified via listed data sources, to be asessed in UK healthcare worker cohort and within listed subgroups, via listed analysis approach in the planned study). For each outcome, multivariable models will incorporate ethnic category and occupational group, and their interactions, adjusted for the four core predictors. The analyses will be conducted by country, and results combined using meta-analysis methods to produce UK estimates. We will report incidence rate ratios, odds ratios, or hazard ratios with 95% confidence intervals and p-values. Forest plots and cumulative incidence curves will be produced. Interactions between ethnicity and other predictors will be presented graphically to aid interpretation. Model assumptions will be assessed.

For peer review only - http://bmjopen.bmj.com/site/about/guidelines.xhtml

We will use directed acyclic graphs to map hypothesised causal associations between ethnicity, occupational group, the core predictors, and COVID-19 outcomes. Mediation analysis methods will then be applied to disentangle the direct, indirect, and mediated interactive effects of ethnic category and COVID-19 outcomes.

HR sub-study

We will conduct a pooled analysis for all countries where available, however where outcomes not available for all countries, country specific results will be presented.

Each outcome will be evaluated using the same regression methods above. Multivariable models will incorporate ethnicity, occupation, and the core predictors as before, whilst extended to incorporate additional predictor information. An ethnicity model will additionally adjust for religion, nationality, and country of birth and incorporate their interactions with ethnicity, if appropriate. An occupation model will additionally adjust for primary area of work, role description, patient/non-patient facing, time in current role, place of work, pay grade, and seniority. Finally, a COVID-19 exposure model will additionally adjust for absence information and total hours worked as time varying exposures, to assess the impact of changes in COVID-19 exposure over time. Forest plots and cumulative incidence curves will be produced. Interactions between ethnicity and other predictors, and the effects of time varying predictors, will be presented graphically to aid interpretation.

Sensitivity and Additional analyses

Dependent on the number of HCWs and events captured, we intend to conduct the following additional analyses:

- **Incorporating BMI and smoking into broad analysis**: adjust for BMI and smoking in COVID-19 outcome models if recorded by EHRs either during, or in the 24 months prior to, the study period.
- **Incorporating grouping by organisation:** incorporate clustering of HCWs within organisations by fitting hierarchical models to account for within organisation correlation and between organisation heterogeneity.
- **Granular analysis within the "Asian/Asian British" ethnicity category**: investigate differences in COVID-19 outcomes between HCWs with South Asian (Indian, Pakistani, and Bangladeshi), East Asian (Chinese), and Other Asian backgrounds.
- **Granular analysis within the "medical staff" occupational group:** investigate differences in COVID-19 outcomes between medical and dental subcategories.
- **Subgroup analysis within hospital workers**: investigate COVID-19 outcomes in hospital workers and cluster HCWs within hospitals using hierarchical models.
- **Subgroup analysis of ancillary workers**: investigate COVID-19 outcomes in ancillary workers through trusts that employ them directly.
- **Missing data:** use multiple imputation to assess the robustness of our findings to missing data.

DISCUSSION AND LIMITATIONS

For peer review only - http://bmjopen.bmj.com/site/about/guidelines.xhtml

As COVID-19 spreads, the number of cases requiring medical intervention will continue. It is crucial to maintain the healthcare system in the UK and control secondary transmission of COVID-19, and measures to identify and protect the HCWs most vulnerable to infection and adverse outcomes must be implemented. However, current COVID-19 risks in HCWs, particularly within those from historically disadvantaged populations, are poorly understood.

Published evidence of COVID-19 outcomes in HCWs are generally from small studies, in single centre or region settings outside the UK, which have not investigated associations with ethnicity [30]. COVID-19 studies which utilise observational data from non-random sampling, such as hospital admissions or voluntary participation, may be affected by collider bias [31]. One such study assesses outcomes using voluntary self-reported data from symptom trackers only [32], thus results are at risk of selection bias due to reliance on accurate self-reporting of exposures and outcomes. A recent study assessing risks of hospitalisation in HCWs and household members in Scotland [18], had insufficient numbers of HCWs from ethnic minority groups to reliably estimate associations between COVID-19 outcomes and ethnicity. This planned study is, to the authors' knowledge, the first UK-wide study to assess the relationship between ethnicity and COVID-19 outcomes in HCWs. This large study incorporates ambitious linkage of multiple anonymised human resource, professional registration, and NHS datasets providing UK-wide coverage of clinical and non-clinical HCWs. However, the lack of a single UK-wide database capturing all HCWs actively working limits our ability to identify all HCWs across all regions. We aim to identify non-clinical ancillary staff not directly employed by the NHS by incorporating contracting organisations.

The broad analysis will examine associations between ethnicity, occupational group, and multiple important COVID-19 outcomes in HCWs employed in the UK, while adjusting for core predictors. Using readily available EHR data enables the investigation of complex models of public health resulting in scalable, population-based measurements of disease burden [33]. However, we foresee a trade-off between the scale and depth of the data, e.g. while incorporating professional registration databases captures HCWs in non-NHS sectors, we are unable to identify whether they are actively working (not retired, on parental or long-term sick leave). Thus, a sub-study will utilise detailed NHS human resource databases to enable granular analyses of ethnicity, occupation, and COVID-19 exposures in those actively working during the pandemic. Additional hypothesis generating analyses will be conducted where interesting results are found, for example if the study identified a higher mortality incidence in a particular ethnic group, a more detailed analysis investigating differences in disease severity by ethnic group could be planned.

Implications regarding the quality of the EHR data have been considered; multiple data sources will be utilised to limit the impact of missing information for the main exposures (ethnicity and occupation) and outcomes (diagnosis, hospitalisation, death). Sensitivity analyses are planned to account for predictors expected to have high proportions of non-random missingness, such as BMI and smoking status. However, this study will be limited by the subset of COVID-19 cases that are diagnosed and recorded within the EHR data. Many HCWs with SARS-CoV-2 infections will not be diagnosed, with a US study projecting up to 63% [28]. Indeed, the study will not capture HCWs with mild or asymptomatic infections in settings where tests are restricted to those with symptoms, and results may be vulnerable to collider bias [31]. Incorporating both suspected and confirmed COVID-19

For peer review only - http://bmjopen.bmj.com/site/about/guidelines.xhtml

diagnoses is likely to increase the number of infections captured, though it is also likely to reduce the specificity of diagnosis by incorporating false positive cases.

Findings from this study will directly inform evidence-based guidance to protect and address health inequalities in HCWs during the ongoing pandemic.

For peer review only

For peer review only - http://bmjopen.bmj.com/site/about/guidelines.xhtml

END MATTER

Funding

This publication presents independent research supported by the MRC-UK Research and Innovation (grant number MR/V027549/1) and National Institute for Health Research (NIHR) rapid response panel to tackle COVID-19, and by the NIHR Leicester Biomedical Research Centre (a partnership between the University of Leicester, University Hospitals of Leicester NHS Trust, and the UK NIHR Applied Research Collaboration East Midlands).

The views and opinions expressed by authors in this publication are those of the authors and do not necessarily reflect those of the NHS, the NIHR, MRC, CCF, NETSCC, or the Department of Health and Social Care.

Author contributions

MPareek is corresponding author for this manuscript and attests that all persons listed as authors meet the ICMJE criteria for authorship.

Funding acquisition: AGuyatt, CJohn, DFord, KAbrams, KKhunti, LGray, MPareek, MTobin.

Conceptualisation: CMelbourne, COrton, DFord, KAbrams, KKhunti, LGray, LTeece, MPareek.

Writing original draft: CMelbourne, COrton, KAbrams, LGray, LTeece.

Revision and editing: AGuyatt, CJohn, CMartin, DFord, DMcAllister, KAbrams, KKhunti, MPareek, MTobin.

Data acquisition, analysis, or interpretation: CMelbourne, COrton, DFord, KAbrams, LGray, LTeece, MPareek.

Statement of competing interests

LTeece is supported by the UK National Institute for Health Research (NIHR) Applied Research Collaboration East Midlands (ARC EM).

LGray leads the NIHR ARC EM Data2Health theme.

KKhunti is Director for the University of Leicester Centre for BME Health, Trustee of the South Asian Health Foundation, national NIHR ARC lead for Ethnicity and Diversity, and a member of Independent SAGE. He is supported by the NIHR ARC EM and the NIHR Leicester Biomedical Research Centre.

CJohn holds a Medical Research Council Clinical Research Training Fellowship (MR/P00167X/1).

KAbrams leads the NIHR ARC EM Data2Health theme, and is supported by Health Data Research UK, the NIHR ARC EM, and as a NIHR Senior Investigator Emeritus (NF-SI-0512-10159). He has served as a paid consultant, providing unrelated methodological and strategic advice, to the pharmaceutical and life sciences industry generally and has received unrelated research funding from Association of the British Pharmaceutical Industry, European Federation of Pharmaceutical Industries & Associations, Pfizer, Sanofi and Swiss Precision Diagnostics. He is a Partner and Director of Visible Analytics Limited, a healthcare consultancy company.

For peer review only

MPareek reports grants and personal fees from Gilead Sciences and personal fees from QIAGEN, outside the submitted work.

Data sharing

As the proposed study involves data on unconsented participants, we will be unable to share individual level data once obtained. Study analysis code will be made available following completion of the study and prior to publication of the study manuscript.

Acknowledgements

This study makes use of anonymised data held in the Secure Anonymised Information Linkage (SAIL) Databank [21]. We would like to acknowledge all the data providers who make anonymised data available for research.

This work uses data provided by participants of the COVID-19 Symptoms Study [22], developed by ZOE Global Limited with scientific and clinical input from King's College London. We would also like to acknowledge all data providers who made anonymised data available for research. We wish to acknowledge the collaborative partnership that enabled acquisition and access to the de-identified data, which led to this output. The collaboration was led by BREATHE, the Health Data Research Hub for Respiratory Health, in partnership with SAIL Databank. We wish to acknowledge the input of ZOE Global Limited and King's College London in their development and sharing of the data, and their input into the understanding and contextualisation of data for COVID-19 research. All research conducted has been completed under the permission and approval of SAIL independent Information Governance Review Panel (IGRP) project number 1120.

The UK-REACH Study Team including: Manish Pareek, Amit Gupta, Anna L. Guyatt, Carl Melbourne, Catherine John, Chris McManus, Chris Orton, David Ford, Edward Dove, Fatimah Wobi, Kamlesh Khunti, Katherine Woolf, Keith R. Abrams, Laura Gray, Laura Nellums, Louise V. Wain, Lucy Teece, Martin Tobin, Mayuri Gogoi, Rob Free, Sue Carr.

For peer review only

REFERENCES

1. WHO, W.H.O., *Coronavirus disease (COVID-19): weekly epidemiological update*. 2020.
2. Chen, N., et al., *Epidemiological and clinical characteristics of 99 cases of 2019 novel coronavirus pneumonia in Wuhan, China: a descriptive study.* The Lancet, 2020. **395**(10223): p. 507-513.
3. Docherty, A.B., et al., *Features of 20 133 UK patients in hospital with covid-19 using the ISARIC WHO Clinical Characterisation Protocol: prospective observational cohort study.* bmj, 2020. **369**.
4. Wynants, L., et al., *Prediction models for diagnosis and prognosis of covid-19 infection: systematic review and critical appraisal.* bmj, 2020. **369**.
5. Yang, J., et al., *Prevalence of comorbidities and its effects in patients infected with SARS-CoV-2: a systematic review and meta-analysis.* International Journal of Infectious Diseases, 2020. **94**: p. 91-95.
6. Patel, A.P., et al., *Race, Socioeconomic Deprivation, and Hospitalization for COVID-19 in English participants of a National Biobank.* medRxiv, 2020.
7. Iacobucci, G., *Covid-19: Deprived areas have the highest death rates in England and Wales*. 2020, British Medical Journal Publishing Group.
8. Aldridge, R.W., et al., *Black, Asian and Minority Ethnic groups in England are at increased risk of death from COVID-19: indirect standardisation of NHS mortality data.* Wellcome Open Research, 2020. **5**(88): p. 88.
9. Harrison, E.M., et al., *Ethnicity and outcomes from COVID-19: the ISARIC CCP-UK prospective observational cohort study of hospitalised patients.* 2020.
10. Mathur, R., et al., *Ethnic differences in COVID-19 infection, hospitalisation, and mortality: an OpenSAFELY analysis of 17 million adults in England.* medRxiv, 2020.
11. Martin, C.A., et al., *Socio-demographic heterogeneity in the prevalence of COVID-19 during lockdown is associated with ethnicity and household size: Results from an observational cohort study.* EClinicalMedicine, 2020: p. 100466.
12. Pan, D., et al., *The impact of ethnicity on clinical outcomes in COVID-19: A systematic review.* EClinicalMedicine, 2020: p. 100404.
13. Chowkwanyun, M. and A.L. Reed Jr, *Racial health disparities and Covid-19—caution and context.* New England Journal of Medicine, 2020.
14. Pareek, M., et al., *Ethnicity and COVID-19: an urgent public health research priority.* The Lancet, 2020. **395**(10234): p. 1421-1422.
15. Lee, C., *"Race" and "ethnicity" in biomedical research: how do scientists construct and explain differences in health?* Social science & medicine, 2009. **68**(6): p. 1183-1190.
16. Khunti, K., et al., *Is ethnicity linked to incidence or outcomes of covid-19?* 2020, British Medical Journal Publishing Group.
17. Koh, D., *Occupational risks for COVID-19 infection.* Occupational medicine (Oxford, England), 2020. **70**(1): p. 3.
18. Shah, A.S., et al., *Risk of hospitalisation with coronavirus disease 2019 in healthcare workers and their households: a nationwide linkage cohort study.* medRxiv, 2020.
19. Cook, T., E. Kursumovic, and S. Lennane, *Exclusive: deaths of NHS staff from covid-19 analysed.* Health Service Journal, 2020. **22**.
20. Levene, L.S., et al., *COVID-19 cumulative mortality rates for frontline healthcare staff in England.* British Journal of General Practice, 2020. **70**(696): p. 327-328.
21. Ford, D.V., et al., *The SAIL Databank: building a national architecture for e-health research and evaluation.* BMC health services research, 2009. **9**(1): p. 157.
22. Drew, D.A., et al., *Rapid implementation of mobile technology for real-time epidemiology of COVID-19.* Science, 2020.

For peer review only - http://bmjopen.bmj.com/site/about/guidelines.xhtml

23. Seidu, S., et al., *The impact of obesity on severe disease and mortality in people with SARS-CoV-2: A systematic review and meta-analysis.* Endocrinology, Diabetes & Metabolism, 2020: p. e00176.
24. Williamson, E., et al., *OpenSAFELY: factors associated with COVID-19-related hospital death in the linked electronic health records of 17 million adult NHS patients.* MedRxiv, 2020.
25. Von Elm, E., et al., *The Strengthening the Reporting of Observational Studies in Epidemiology (STROBE) statement: guidelines for reporting observational studies.* Annals of internal medicine, 2007. **147**(8): p. 573-577.
26. Nicholls, S.G., et al., *The reporting of studies conducted using observational routinely-collected health data (RECORD) statement: methods for arriving at consensus and developing reporting guidelines.* PLoS One, 2015. **10**(5): p. e0125620.
27. ONS, O.f.N.S., *Coronavirus (COVID-19) Infection Survey pilot: England, 31 July 2020*. 2020.
28. Gao, R. and K.H. Rosenlof, *Mortality rate and estimate of fraction of undiagnosed COVID-19 cases in the US in March and April 2020.* medRxiv, 2020.
29. Verity, R., et al., *Estimates of the severity of coronavirus disease 2019: a model-based analysis.* The Lancet infectious diseases, 2020.
30. Chou, R., et al., *Epidemiology of and risk factors for coronavirus infection in health care workers: a living rapid review.* Annals of internal medicine, 2020.
31. Griffith, G., et al., *Collider bias undermines our understanding of COVID-19 disease risk and severity.* medRxiv, 2020.
32. Nguyen, L.H., et al., *Risk of COVID-19 among front-line health-care workers and the general community: a prospective cohort study.* The Lancet Public Health, 2020.
33. Hemingway, H., et al., *Big data from electronic health records for early and late translational cardiovascular research: challenges and potential.* European heart journal, 2018. **39**(16): p. 1481-1495.
34. Menni, C., et al., *Real-time tracking of self-reported symptoms to predict potential COVID-19.* Nature medicine, 2020: p. 1-4.

For peer review only

TABLES

Table 1: Coverage of human resources and professional registration databases utilised to identifiy UK health care worker cohort for the planned study

Registry	Approximate Size	Country Coverage	Occupational coverage
**Electronic Staff Record (ESR)**	1.4 million	England[1] and Wales	Staff directly employed by NHS England and Wales.
**Scottish Workforce Information Standard System (SWISS)**	163K	Scotland	Staff directly employed by NHS Scotland.
**General Medical Council (GMC)**	336k	England, Wales, Scotland and Northern Ireland	All doctors[2].
**Nursing & Midwifery Council (NMC)**	706k	England, Wales, Scotland and Northern Ireland	Nurses, midwives, student nurses and nursing associates[3].
**General Dental Council (GDC)**	110k	England, Wales, Scotland and Northern Ireland	Dentists, dental care professionals[4] and dental practices.
**General Optical Council (GOC)**	30k	England, Wales, Scotland and Northern Ireland	Optometrists, dispensing opticians, student opticians.
**General Pharmaceutical Council (GPhC)**	94k	England, Wales and Scotland	Pharmacists and pharmacy technicians.
**Pharmaceutical Society Northern Ireland (PSNI)**	3k	Northern Ireland	Pharmacist and trainee pharmacists.
**Health and Care Professions Council (HCPC)**	280k	England, Wales, Scotland, Northern Ireland, and international[5]	Art therapists, biomedical scientists, chiropodists, podiatrists, clinical scientists, dietitians, hearing aid dispensers, occupational therapists, operating department practitioners, orthoptists, paramedics, physiotherapists, practitioner psychologists, prosthetists, orthotists, radiographers, speech and language therapists.

[1]Two foundation trusts in England do not currently use ESR.
[2]Including those in UK Foundation Year 1 and 2 posts, general practitioners, and specialist consultants.
[3]In England only.
[4]Dental care professionals include clinical dental technicians, dental hygienists, dental nursed, dental technicians, dental therapists, orthodontic therapists.
[5]International professionals will not be included in this study as linkage to EHRs, thus assessment of outcomes, will not be possible.

For peer review only

Table 2: ONS ethnicity groupings

Broad ethnic categories	Ethnic groups
**White**	English/Welsh/Scottish/Northern Irish/British
	Irish
	Gypsy or Irish Traveller
	Any other White background
**Mixed/Multiple ethnic groups**	White and Black Caribbean
	White and Black African
	White and Asian
	Any other Mixed/Multiple ethnic background
**Asian/Asian British**	Indian
	Pakistani
	Bangladeshi
	Chinese
	Any other Asian background
**Black/African/Caribbean/Black British**	African
	Caribbean
	Any other Black/African/Caribbean background
**Other ethnic group**	Arab
	Any other ethnic group

For peer review only

Table 3 : COVID-19 outcomes and definitions, identified via listed data sources, to be asessed in UK healthcare worker cohort and within listed subgroups, via listed analysis approach in the planned study

**Outcomes**		**Definitions**	**Data sources**	**Subgroups**	**Analysis approach (output)**
**COVID-19 diagnosis**	Confirmed COVID-19 diagnosis	A positive swab test for SARS-CoV-2 during the study period	PHE Pillar 1 (PHE labs or NHS hospital) and Pillar 2 (wider population) testing data[1]		Poisson regression (IRR)
	Suspected COVID-19 diagnosis	Self-reporting of either a positive SARS-CoV-2 test or combination of symptoms shown to be predictive of positive testing[2] during the study period or suspected case SNOMED CT codes (NHS Digital) recorded during the study period	COVID-19 Symptom Study app and primary care data		Poisson regression (IRR)
**Hospitalisation**	All hospitalisations	Any hospital admission recorded during the study period	Secondary care and CHESS data	Confirmed COVID-19 diagnosis	Logistic regression (OR)
	COVID-19 hospitalisations	hospital admissions with COVID-19 specific ICD-10 codes[3] during the study period or positive swab test for SARS-CoV-2 recorded during or within 28 days prior to admission	Secondary care, CHESS, PHE Pillar 1 (PHE labs or NHS hospital) and Pillar 2 (wider population) testing data[1]	Confirmed COVID-19 diagnosis	Logistic regression (OR)
	COVID-19 ITU admissions	Admissions to ITU during the study period	Secondary care, CHESS, and intensive care audit data	Confirmed COVID-19 diagnosis	Logistic regression (OR)
**Mortality**	All-cause mortality	Any death registration recorded during the study period	ONS mortality, primary care, and secondary care data	Confirmed COVID-19 diagnosis<br>COVID-19 hospitalisations<br>Covid-19 ITU admissions	Flexible parametric time-to-event regression (HR)
	Mortality due to COVID-19	any death registration with COVID-19 specific ICD-10 codes[3] recorded for the primary or secondary cause of death during the study period	ONS mortality, primary care, and secondary care data	Confirmed COVID-19 diagnosis<br>COVID-19 hospitalisations<br>Covid-19 ITU admissions	Flexible parametric time-to-event regression (HR)

For peer review only - http://bmjopen.bmj.com/site/about/guidelines.xhtml

COVID-19, Coronavirus disease 2019; SARS-CoV-2, severe acute respiratory syndrome coronavirus 2; PHE, Public Health England; NHS, National Health Service; IRR, incidence rate ratio; SNOMED CT, Systematized Nomenclature of Medicine - Clinical Terms; CHESS, COVID-19 Hospitalisation in England Surveillance System; OR, odds ratio; ICD-10, International Classification of Diseases – 10th revision; ITU, Intensive Therapy Unit; ONS, Office for National Statistics; HR, hazard ratio.
[1]PHE Pillar 3 (serology testing for antibodies) and Pillar 4 (blood and swab testing) data will be incorporated if obtained.
[2]Symptoms include loss of smell or taste, fatigue, persistent cough, and loss of appetite [34].
[3]COVID-19 specific ICD-10 codes are U07.1: "COVID-19, virus identified" and U07.2: "COVID-19, virus not identified".

For peer review only

For peer review only - http://bmjopen.bmj.com/site/about/guidelines.xhtml

Table 4 : Definitions for core predictors associated with risk of adverse COVID-19 outcomes

Predictor	Definition
**Age**	A continuous measure of age (in years) at the index date (01-02-2020), calculated using date of birth as recorded in HR or professional registration databases.
**Sex**	A categorical measure (female/male/other) as recorded in HR or professional registration databases.
**Comorbidities**	A categorical measure counting the number of comorbidities for each individual (0, 1, or 2 or more) obtained from primary care records and supplemented with secondary care records in analyses conducted in the hospitalised cohort. Comorbidity count will incorporate conditions from the NHS Digital shielded patient list algorithm and others found to be associated with adverse COVID-19 outcomes.
**Socio-economic deprivation**	An ordinal measure using quintiles of the Index of Multiple Deprivation (IMD), the official measure of relative deprivation for small areas in each country, based on residential postcode as recorded in HR or professional registration databases. Where residential postcode is not provided workplace postcode will be used as a surrogate.

For peer review only

FIGURES

The above diagram and style was interpreted from an initial data flow diagram created and provided by Andy Boyd at the University of Bristol. It has been repurposed and amended to illustrate data flows specific to the UK-REACH project by Chris Orton at Swansea University.

Figure 1 : Data flow and linkage of UK-REACH data sources

For peer review only

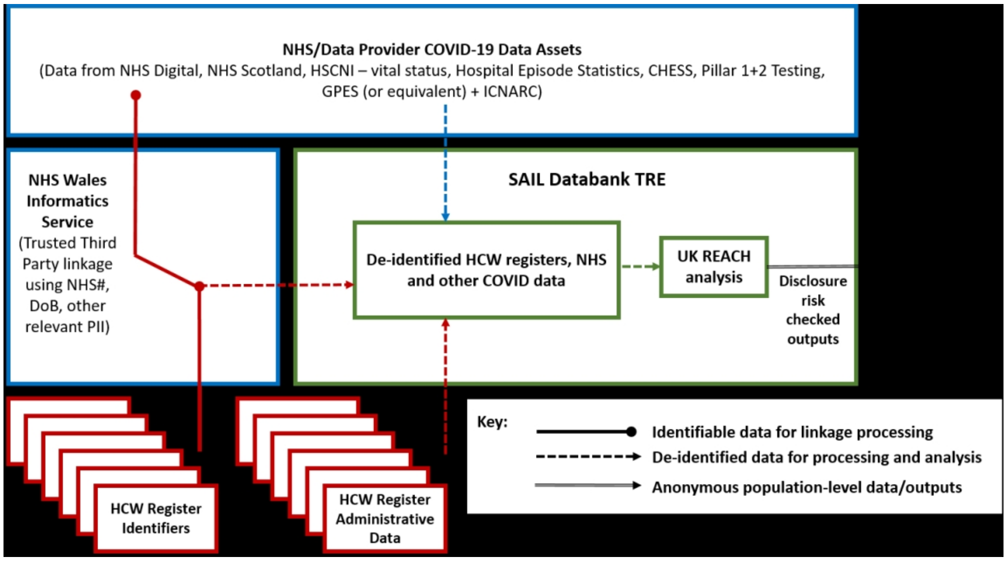

Data flow and linkage of UK-REACH data sources.
The above diagram and style was interpreted from an initial data flow diagram created and provided by Andy Boyd at the University of Bristol. It has been repurposed and amended to illustrate data flows specific to the UK-REACH project by Chris Orton at Swansea University.

The RECORD statement – checklist of items, extended from the STROBE statement, that should be reported in observational studies using routinely collected health data.

	Item No.	STROBE items	Location in manuscript where items are reported	RECORD items	Location in manuscript where items are reported
**Title and abstract**					
	1	(a) Indicate the study's design with a commonly used term in the title or the abstract (b) Provide in the abstract an informative and balanced summary of what was done and what was found	Title – P1<br>Abstract – P2	RECORD 1.1: The type of data used should be specified in the title or abstract. When possible, the name of the databases used should be included.<br><br>RECORD 1.2: If applicable, the geographic region and timeframe within which the study took place should be reported in the title or abstract.<br><br>RECORD 1.3: If linkage between databases was conducted for the study, this should be clearly stated in the title or abstract.	Title – P1<br>Abstract – P2<br><br>Abstract – P2<br><br>Title – P1
**Introduction**					
Background rationale	2	Explain the scientific background and rationale for the investigation being reported	Introduction – P4		
Objectives	3	State specific objectives, including any prespecified hypotheses	Objectives – P5		
**Methods**					
Study Design	4	Present key elements of study design early in the paper	Study design – P6		
Setting	5	Describe the setting, locations, and relevant dates, including periods of recruitment, exposure, follow-up, and data collection	Data sources – P6-7<br>Observation period – P7		

For peer review only

Participants	6	*(a) Cohort study* - Give the eligibility criteria, and the sources and methods of selection of participants. Describe methods of follow-up<br>*Case-control study* - Give the eligibility criteria, and the sources and methods of case ascertainment and control selection. Give the rationale for the choice of cases and controls<br>*Cross-sectional study* - Give the eligibility criteria, and the sources and methods of selection of participants<br><br>*(b) Cohort study* - For matched studies, give matching criteria and number of exposed and unexposed<br>*Case-control study* - For matched studies, give matching criteria and the number of controls per case	Study population – P7<br><br>Observational period – P7<br><br>NA	RECORD 6.1: The methods of study population selection (such as codes or algorithms used to identify subjects) should be listed in detail. If this is not possible, an explanation should be provided.<br><br>RECORD 6.2: Any validation studies of the codes or algorithms used to select the population should be referenced. If validation was conducted for this study and not published elsewhere, detailed methods and results should be provided.<br><br>RECORD 6.3: If the study involved linkage of databases, consider use of a flow diagram or other graphical display to demonstrate the data linkage process, including the number of individuals with linked data at each stage.	Study population – P7<br><br>NA<br><br>Figure1
Variables	7	Clearly define all outcomes, exposures, predictors, potential confounders, and effect modifiers. Give diagnostic criteria, if applicable.	Main exposures – P8<br>Outcomes – P8 & Table 3<br>Predictors – P8 & Table 4	RECORD 7.1: A complete list of codes and algorithms used to classify exposures, outcomes, confounders, and effect modifiers should be provided. If these cannot be reported, an explanation should be provided.	Main exposures – P8<br>Outcomes – P8 & Table 3<br>Predictors – P8 & Table 4
Data sources/ measurement	8	For each variable of interest, give sources of data and details of methods of assessment (measurement).<br>Describe comparability of assessment methods if there is more than one group	Data sources – P6-7<br>Main exposures – P8<br>Outcomes – P8 & Table 3<br>Predictors – P8 & Table 4		

For peer review only

Bias	9	Describe any efforts to address potential sources of bias	Discussion – P13		
Study size	10	Explain how the study size was arrived at	Sample size – P10		
Quantitative variables	11	Explain how quantitative variables were handled in the analyses. If applicable, describe which groupings were chosen, and why	Main exposures – P8<br>Predictors – P8 & Table 4		
Statistical methods	12	(a) Describe all statistical methods, including those used to control for confounding<br>(b) Describe any methods used to examine subgroups and interactions<br>(c) Explain how missing data were addressed<br>(d) *Cohort study* - If applicable, explain how loss to follow-up was addressed<br>*Case-control study* - If applicable, explain how matching of cases and controls was addressed<br>*Cross-sectional study* - If applicable, describe analytical methods taking account of sampling strategy<br>(e) Describe any sensitivity analyses	Statistical analysis plan – P10-12<br><br>Sensitivity and Additional analyses – P12<br>Missing data – P10-11<br>HR sub-study – P12<br><br>Sensitivity and Additional analyses – P12		
Data access and cleaning methods		..		RECORD 12.1: Authors should describe the extent to which the investigators had access to the database population used to create the study population.	Data linkage – P7 & Figure 1

For peer review only

				RECORD 12.2: Authors should provide information on the data cleaning methods used in the study.	N/A - protocol
Linkage		..		RECORD 12.3: State whether the study included person-level, institutional-level, or other data linkage across two or more databases. The methods of linkage and methods of linkage quality evaluation should be provided.	Data linkage – P7
**Results**					
Participants	13	(a) Report the numbers of individuals at each stage of the study (*e.g.*, numbers potentially eligible, examined for eligibility, confirmed eligible, included in the study, completing follow-up, and analysed)<br>(b) Give reasons for non-participation at each stage.<br>(c) Consider use of a flow diagram	N/A - protocol<br>N/A - protocol<br>N/A - protocol	RECORD 13.1: Describe in detail the selection of the persons included in the study (*i.e.,* study population selection) including filtering based on data quality, data availability and linkage. The selection of included persons can be described in the text and/or by means of the study flow diagram.	N/A - protocol
Descriptive data	14	(a) Give characteristics of study participants (*e.g.*, demographic, clinical, social) and information on exposures and potential confounders<br>(b) Indicate the number of participants with missing data for each variable of interest<br>(c) *Cohort study* - summarise follow-up time (*e.g.*, average and total amount)	N/A - protocol<br>N/A - protocol<br>N/A - protocol		
Outcome data	15	*Cohort study* - Report numbers of outcome events or summary measures over time<br>*Case-control study* - Report numbers in each exposure	N/A - protocol		

For peer review only

		category, or summary measures of exposure<br>*Cross-sectional study* - Report numbers of outcome events or summary measures			
Main results	16	(a) Give unadjusted estimates and, if applicable, confounder-adjusted estimates and their precision (e.g., 95% confidence interval). Make clear which confounders were adjusted for and why they were included<br>(b) Report category boundaries when continuous variables were categorized<br>(c) If relevant, consider translating estimates of relative risk into absolute risk for a meaningful time period	N/A - protocol<br>N/A - protocol<br>N/A - protocol		
Other analyses	17	Report other analyses done—e.g., analyses of subgroups and interactions, and sensitivity analyses	N/A - protocol		
**Discussion**					
Key results	18	Summarise key results with reference to study objectives	N/A - protocol		
Limitations	19	Discuss limitations of the study, taking into account sources of potential bias or imprecision. Discuss both direction and magnitude of any potential bias	Discussion and limitations -P12-13	RECORD 19.1: Discuss the implications of using data that were not created or collected to answer the specific research question(s). Include discussion of misclassification bias, unmeasured confounding, missing data, and changing eligibility over time, as they pertain to the study being reported.	Discussions and limitation - P12
Interpretation	20	Give a cautious overall interpretation of results considering objectives,	N/A - protocol		

		limitations, multiplicity of analyses, results from similar studies, and other relevant evidence			
Generalisability	21	Discuss the generalisability (external validity) of the study results	Discussion and limitations -P12-13		
**Other Information**					
Funding	22	Give the source of funding and the role of the funders for the present study and, if applicable, for the original study on which the present article is based	Funding statement P14		
Accessibility of protocol, raw data, and programming code		..		RECORD 22.1: Authors should provide information on how to access any supplemental information such as the study protocol, raw data, or programming code.	Data sharing – P14

*Reference: Benchimol EI, Smeeth L, Guttmann A, Harron K, Moher D, Petersen I, Sørensen HT, von Elm E, Langan SM, the RECORD Working Committee. The REporting of studies Conducted using Observational Routinely-collected health Data (RECORD) Statement. *PLoS Medicine* 2015; in press.

*Checklist is protected under Creative Commons Attribution (CC BY) license.

For peer review only

BMJ Open

United Kingdom Research study into Ethnicity And COVID-19 outcomes in Healthcare workers (UK-REACH): a retrospective cohort study using linked routinely collected data, study protocol

Journal:	*BMJ Open*
Manuscript ID	bmjopen-2020-046392.R1
Article Type:	Protocol
Date Submitted by the Author:	03-Mar-2021
Complete List of Authors:	Teece, Lucy; University of Leicester, Biostatistics Research Group, Department of Health Sciences<br>Gray, Laura; University of Leicester, Biostatistics Research Group, Department of Health Sciences<br>Melbourne, Carl; University of Leicester, Genetic Epidemiology Research Group, Department of Health Sciences<br>Orton, Chris; Swansea University Medical School, Population Data Science<br>Ford, David; Swansea University Medical School, Population Data Science<br>Martin, Christopher; University of Leicester, Department of Respiratory Sciences; University Hospitals of Leicester NHS Trust, Department of Infection and HIV Medicine<br>McAllister, David; University of Glasgow, Institute of Health and Wellbeing<br>Khunti, Kamlesh; Leicester General Hospital, Diabetes Research Centre, University of Leicester; Leicester General Hospital, Leicester Real World Evidence Unit, University of Leicester<br>Tobin, Martin; University of Leicester, Genetic Epidemiology Research Group, Department of Health Sciences; NIHR Leicester Biomedical Research Centre Respiratory Diseases, Glenfield Hospital<br>John, Catherine; University of Leicester, Genetic Epidemiology Research Group, Department of Health Sciences<br>Abrams, Keith; University of York, Centre for Health Economics<br>Pareek, Manish; University of Leicester, Department of Respiratory Sciences; University Hospitals of Leicester NHS Trust, Department of Infection and HIV Medicine
**Primary Subject Heading**:	Public health
Secondary Subject Heading:	Infectious diseases
Keywords:	COVID-19, EPIDEMIOLOGY, Public health < INFECTIOUS DISEASES, Adult intensive & critical care < INTENSIVE & CRITICAL CARE

I, the Submitting Author has the right to grant and does grant on behalf of all authors of the Work (as defined in the below author licence), an exclusive licence and/or a non-exclusive licence for contributions from authors who are: i) UK Crown employees; ii) where BMJ has agreed a CC-BY licence shall apply, and/or iii) in accordance with the terms applicable for US Federal Government officers or employees acting as part of their official duties; on a worldwide, perpetual, irrevocable, royalty-free basis to BMJ Publishing Group Ltd ("BMJ") its licensees and where the relevant Journal is co-owned by BMJ to the co-owners of the Journal, to publish the Work in this journal and any other BMJ products and to exploit all rights, as set out in our licence.

The Submitting Author accepts and understands that any supply made under these terms is made by BMJ to the Submitting Author unless you are acting as an employee on behalf of your employer or a postgraduate student of an affiliated institution which is paying any applicable article publishing charge ("APC") for Open Access articles. Where the Submitting Author wishes to make the Work available on an Open Access basis (and intends to pay the relevant APC), the terms of reuse of such Open Access shall be governed by a Creative Commons licence – details of these licences and which Creative Commons licence will apply to this Work are set out in our licence referred to above.

Other than as permitted in any relevant BMJ Author's Self Archiving Policies, I confirm this Work has not been accepted for publication elsewhere, is not being considered for publication elsewhere and does not duplicate material already published. I confirm all authors consent to publication of this Work and authorise the granting of this licence.

For peer review only

United Kingdom Research study into Ethnicity And COVID-19 outcomes in Healthcare workers (UK-REACH): a retrospective cohort study using linked routinely collected data, study protocol

Lucy Teece[1], Laura J Gray[1], Carl Melbourne[2], Chris Orton[3], David V Ford[3], Christopher A Martin[4,5], David A McAllister[6], Kamlesh Khunti[7,8], Martin Tobin[2,9], Catherine John[2], Keith R Abrams[10], Manish Pareek[4,5]

For the UK-REACH Study Collaborative Group.

[1]Biostatistics Research Group, Department of Health Sciences, University of Leicester, Leicester, UK.

[2]Genetic Epidemiology Research Group, Department of Health Sciences, University of Leicester, Leicester, UK.

[3]Population Data Science, Swansea University Medical School, Swansea University, Swansea, UK.

[4]Department of Respiratory Sciences, University of Leicester, Leicester, UK.

[5]Department of Infection and HIV Medicine, University Hospitals of Leicester NHS Trust, Leicester, UK.

[6]Institute of Health and Wellbeing, University of Glasgow, Glasgow, UK.

[7]Diabetes Research Centre, University of Leicester, Leicester General Hospital, Leicester, UK.

[8]Leicester Real World Evidence Unit, University of Leicester, Leicester General Hospital, Leicester, UK.

[9]National Institute for Health Research, Leicester Respiratory Biomedical Research Centre, Glenfield Hospital, Leicester, UK

[10]Centre for Health Economics, University of York, York, UK.

Key words: COVID-19, diagnosis, hospitalisation, mortality, healthcare workers, ethnicity.

Corresponding author (& contact info): Manish Pareek (manish.pareek@leicester.ac.uk)

Word count main text: 4,349

Word count abstract: 234

For peer review only - http://bmjopen.bmj.com/site/about/guidelines.xhtml

ABSTRACT

Introduction;

Coronavirus disease 2019 (COVID-19) has spread rapidly worldwide, causing significant morbidity and mortality. People from ethnic minorities, particularly those working in healthcare settings, have been disproportionately affected. Current evidence of the association between ethnicity and COVID-19 outcomes in people working in healthcare settings is insufficient to inform plans to address health inequalities.

Methods and analysis;

This study combines anonymised human resource databases with professional registration and NHS datasets to assess associations between ethnicity and COVID-19 diagnosis, hospitalisation, and death in healthcare workers in the UK. Adverse COVID-19 outcomes will be assessed between 01-02-2020 (date following first confirmed COVID-19 case in UK) and study end date (31-01-2021), allowing a maximum of one year follow-up.

Planned analyses include multivariable Poisson, logistic, and flexible parametric time-to-event regression within each country, adjusting for core predictors, followed by meta-analysis of country-specific results to produce combined effect estimates for the UK. Mediation analysis methods will be explored to examine the direct, indirect, and mediated interactive effects between ethnicity, occupational group, and COVID-19 outcomes.

Ethics and dissemination;

Ethical approval for the UK-REACH programme has been obtained via the expedited HRA COVID-19 processes (REC ref: 20/HRA/4718, IRAS ID: 288316). Research information will be anonymised via the Secure Anonymised Information Linkage (SAIL) Databank before release to researchers. Study results will be submitted for publication in an open access peer-reviewed journal and made available on our dedicated website (https://uk-reach.org/).

ISRCTN Registration number;

ISRCTN11811602

For peer review only

ARTICLE SUMMARY

Strengths and limitations of this study

1. First large UK-wide study assessing the associations between ethnicity and COVID-19 outcomes in people who work in healthcare settings.
2. Ambitious linkage of multiple anonymised human resource and professional registration databases with NHS datasets, aiming to provide coverage of the whole of the UK.
3. Broad and comprehensive analysis that aims to unpick the associations between ethnicity, occupational group, and multiple COVID-19 outcomes while adjusting for core predictors.
4. Access to highly detailed human resource databases (including Electronic Staff Record for England and Wales) enables granular analyses of ethnicity, occupation, and COVID-19 exposures.
5. While every effort has been made to identify as many health care workers as possible, the lack of a single UK-wide database capturing those actively working within healthcare settings is a key limitation that may reduce our ability to identify all health care workers across all regions.

For peer review only

For peer review only - http://bmjopen.bmj.com/site/about/guidelines.xhtml

INTRODUCTION

Coronavirus disease 2019 (COVID-19) has been declared a global pandemic. Since the first reported cases in December 2019, over 110.7 million cases and 2.4 million deaths have been reported worldwide [1]. Consistent evidence identifies advanced age, male sex, socio-economic deprivation, and chronic comorbidities as core predictors for COVID-19 adverse outcomes [2-5].

The COVID-19 pandemic has disproportionately impacted historically disadvantaged populations [6]. In the UK, higher infection and mortality rates are observed in areas with greater deprivation [7] and amongst ethnic minorities [8-12]. This has prompted calls to prioritise the publication of data disaggregated by ethnicity and to conduct research which increases our understanding of risk in these populations [13, 14]. Ethnicity is a complex construct which represents social, biological, or genetic differences between populations [15]. Potential explanations for COVID-19 related health disparities between ethnic groups include socioeconomic, cultural, behavioural, biological, or pathophysiological differences [16]. It is recommended that investigations into ethnicity-related health inequality should also consider interrelated factors such as deprivation, religion, and pre-existing conditions. This approach serves to highlight the interplay between ethnic disparities and economic inequality [13] and disentangle the independent importance of these factors [14].

Those working in healthcare settings, hereafter referred to as Health Care Workers (HCWs), have a greater risk of severe acute respiratory syndrome coronavirus 2 (SARS-CoV-2) exposure and transmission due to the number of COVID-19 cases requiring medical intervention [17]. A Scottish linkage study concluded over a sixth of hospitalised COVID-19 cases were HCWs or members of their households [18]. There is concern that HCWs from ethnic minority groups are at increased risk of adverse outcomes from COVID-19. However, the interplay between ethnicity and healthcare occupation and its impact on COVID-19 outcomes is unclear. Publicly available data indicates that individuals from ethnic minorities account for 65-76% of deaths reported in clinical HCWs, despite contributing less than 20% of the NHS workforce [19, 20]. We must understand differences in risks in these populations to protect the most vulnerable HCWs and maintain a functioning healthcare system [18]. However, the quality of data available to evaluate COVID-19 outcomes in HCWs, and the impact of ethnicity on these outcomes, remains poor and limited by small sample size.

The United Kingdom Research study into Ethnicity And COVID-19 diagnosis and outcomes in Healthcare workers (UK-REACH) programme will rapidly examine differences in COVID-19 diagnosis, clinical outcomes, professional practices, and well-being among HCWs from different ethnicities. It will provide rapid evidence through five interlinked work packages; a large data linkage cohort study, a longitudinal cohort study, a legal/ethical analysis, a qualitative work stream, and the development of a stakeholder working group. Here we describe the protocol for the large data linkage cohort study, which will bring together anonymised human resource, professional registration, and NHS datasets within a Trusted Research Environment [21]. This linked database will be used to assess the relationship between ethnicity and COVID-19 diagnosis, hospitalisation, and death in HCWs.

Objectives

Our primary aim is to determine whether COVID-19 diagnosis, hospitalisation, and mortality rates differ between ethnic and occupational groups in HCWs. We will conduct both a broad analysis,

For peer review only - http://bmjopen.bmj.com/site/about/guidelines.xhtml

encompassing all those registered as HCWs on 01-02-2020 in the UK, and a detailed sub-study analysis in those actively working during the COVID-19 pandemic, incorporating more granular ethnicity, occupation, and exposure information.

Considering current evidence, we hypothesise that HCWs from ethnic minority backgrounds will have increased risk of COVID-19 diagnosis and adverse outcomes compared to their White counterparts regardless of occupation.

For peer review only

For peer review only - http://bmjopen.bmj.com/site/about/guidelines.xhtml

METHODS

Study design

We will utilise data from multiple linked electronic health record (EHR) databases to investigate the study hypotheses within a cohort of HCWs. The cohort study will begin on 01-02-2020 (date following first confirmed COVID-19 case in UK) and follow participants for one year, until 31-01-2021.

UK healthcare data sources

For all residents in the UK, healthcare is free at the point of delivery as part of the Government-funded National Health Service (NHS). The NHS employs around 1.6 million people in hospital and community health services; over half are professionally qualified clinical staff (including doctors, nurses, midwives, technical and ambulance staff), the remainder support clinical staff and the NHS Infrastructure. However, identification of all healthcare workers in the UK is not straightforward. There is no single UK-wide database capturing these employees as the health systems in England, Scotland, Wales, and Northern Ireland have diverged since political devolution in 1999. Further, many staff groups that provide NHS services, including general practitioners and dentists, are not directly employed by the NHS.

Our primary data sources for identifying healthcare workers are the NHS human resource (HR) and payroll databases, namely the Electronic Staff Record (ESR), capturing approximately 1.4 million staff paid through the NHS in England and Wales, and the Scottish Workforce Information Standard System (SWISS) including approximately 170,000 staff directly employed by NHS Scotland. As well as clinical staff, these databases include NHS paid ancillary and administrative services staff, and those in management roles. These HR databases cover NHS organisations including hospital trusts, ambulance services, and clinical commissioning groups with comprehensive employment data during the cohort period of interest (Table 1). We also aim to access HR data from non-NHS ancillary worker contractor companies to capture non-clinical staff and those working in facilities management and care coordination positions.

To enable wider coverage of the UK and capture workers in non-NHS sectors (e.g. community and primary care), the HR data will be supplemented by health and social care regulators, where available. These include the General Medical Council (GMC), Nursing and Midwifery Council (NMC), General Dental Council (GDC), General Pharmaceutical Council (GPhC), General Optical Council (GOC), Pharmaceutical Society Northern Ireland (PSNI), and Health and Care Professions Council (HCPC). Where possible, we will obtain data for all HCWs registered to these regulators on the cohort inception date (01-02-2020). As these registries provide less information on specific occupational role and workplace location, it's not possible to identify those actively working during the study period. All included bodies provide UK-wide coverage, except pharmaceutical bodies GPhC and PSNI, which regulate Great Britain and Northern Ireland separately. Table 1 compares the coverage, inclusion, and data available from each registration database.

Given the differences in coverage and data across databases, the analysis will be two-fold. Firstly, a broad analysis will address the main objective using the full UK-wide cohort for any HCW employed or registered on 01-02-2020. Secondly, a detailed HR sub-study will incorporate additional information

For peer review only

on ethnicity, occupational role, and potential for SARS-CoV-2 exposure (including whether an individual actively worked during the study period), and changes to these throughout the study period.

COVID-19 diagnosis, hospitalisation, and mortality data will be gathered from several routinely collected EHR databases. We aim to obtain primary and secondary care datasets as well as specific intensive care audit data (such as Intensive Care National Audit & Research Centre data for England and Wales and Scottish Intensive Care Society Audit Group data for Scotland) and mortality information (Office for National Statistics, ONS) for each country. We also plan to incorporate specialised COVID-19 related databases, including COVID-19 surveillance data (COVID-19 Hospitalisation in England Surveillance System, CHESS), COVID Symptom Study app data [22], and Public Health England (PHE) Pillar 1 (within hospital) and Pillar 2 (community-based) testing data, where available. Additional datasets, such as PHE Pillar 3 and 4, will be sought and incorporated if obtained.

It may not be possible to access all outcome datasets for England, Wales, Scotland, and Northern Ireland. Therefore, outcomes will be assessed separately in each country and results combined using meta-analysis methods to produce UK-wide estimates.

Data linkage

Anonymised data will be stored at the Secure Anonymised Information Linkage (SAIL) databank [21] SAIL works in partnership with a trusted third party, the NHS Wales Informatics Service (NWIS), to ensure processing of personal data is kept within the NHS and separate from the research environment. The process for anonymization and linkage is depicted in Figure 1.

To process to create the cohort of healthcare workers is as follows: Information from the HR and regulator databases will be separated into identifiable data and other administrative data. The identifiable data will be sent directly to NWIS and anonymised by encrypting identifiers into a single anonymous ID. Databases will be linked using this ID to identify the study cohort of healthcare workers and duplicate information from individuals present in more than one of the HR and regulator databases will be removed. Other administrative data will be sent directly to SAIL, who will use the anonymous study ID (subsequently removed to prohibit reverse engineering of re-identification) to match to the anonymised data.

The process for obtaining linked healthcare records for the cohort is as follows: NWIS will share the identifiers from the study cohort with the relevant health record providers, such as NHS Digital, NHS Scotland, and the Northern Ireland Honest Broker Service, to establish linkage to required datasets. The health record providers then return the linked health records, including information for outcomes and predictors, following the same split-file process (identifiable data to NWIS, anonymised data to SAIL). Identifiable health record data will be kept entirely within the NHS. Linked anonymised records will be matched in SAIL using the anonymous study ID to enable the project analysis. Once the linkage process is complete, the identifiable data will be deleted in accordance with the contractual arrangement between NWIS and SAIL.

We will develop a process to enable access to the UK-REACH data for other researchers once this study is complete. This process will engage with the above health record providers to establish onward sharing in partnership with the SAIL Databank.

For peer review only - http://bmjopen.bmj.com/site/about/guidelines.xhtml
For peer review only

Study population

The cohort will include all adult (aged 16 years and over) HCWs captured by HR records or registered with at least one professional regulator on 01-02-2020, excluding those which linkage to EHRs is unsuccessful. HCWs with missing ethnicity, occupation, age, sex, or postcode will be excluded from the main analysis. For the HR sub-study, only those actively working will be included, thus excluding those on parental, sickness, or disability leave for the duration of study period.

Observation period

Follow-up commences on the index date (01-02-2020) (or date of return/commencement of employment for the HR sub-study) and continue until either their death date (COVID-19 related or due to other causes) or the study end date (31-01-2021). For the HR sub-study, those leaving NHS employment during the study period will be included up until this date, at which point they will be censored.

Main exposures

The main exposure of interest is ethnicity, which is likely to be inconsistently recorded across databases. We will primarily assign ethnicity using staff HR records (ESR or SWISS) and registration databases (if not included/missing from HR records). If ethnicity is missing from these databases, linked EHRs will be assessed. Those with ethnicity recorded as 'not stated' or with completely missing ethnicity information will be excluded from the main analyses. Ethnicity will be categorised based on ONS groupings (Table 2).

A more granular analysis will incorporate subdivisions of ethnicity (particularly within Asian/Asian British category) if a sufficient number of events are observed.

Occupational group (and its interaction with ethnicity) is also of primary interest. We will assign occupation for the main cohort using data recorded on 01-02-2020 from either HR records or data held by the regulating bodies and the following categories informed by version 17.1 of the NHS Occupational Code Manual (*NHS Digital*):

- Ambulance staff
- Administrations and estates staff
- Health care assistants and other support staff
- Medical staff
- Dental staff
- Nursing, midwifery and health visiting staff
- Nursing, midwifery and health visiting learners
- Scientific, therapeutic and technical staff
- Healthcare sciences
- General payments

A more granular analysis will incorporate subdivisions of occupational groups (particularly the Medical staff group) if a sufficient number of events are observed.

Outcomes

For peer review only - http://bmjopen.bmj.com/site/about/guidelines.xhtml

The main outcomes are COVID-19 diagnosis (confirmed and suspected, confirmed only), hospitalisation (all, COVID-19 specific, ITU admission) and mortality (all-cause, COVID-19 specific). The definitions, data sources, and a summary of planned analyses for these outcomes are provided in Table 3 : COVID-19 outcomes and definitions, identified via listed data sources, to be asessed in UK healthcare worker cohort and within listed subgroups, via listed analysis approach in the planned study.

Predictors

We will investigate and adjust for the following core predictors: age, sex, comorbidities, and socio-economic deprivation. A description of the definitions for these core predictors, and how they will be assessed, is provided in Table 4.

Elevated body mass index (BMI), obesity, and smoking are also considered key predictors for adverse COVID-19 outcomes [9, 23, 24] however are likely to be missing for large proportions of the identified cohort. Furthermore, missingness in EHR data is unlikely to be random, preventing standard imputation approaches. Thus, BMI and smoking will not be included as independent predictors in the primary study but instead in a sensitivity analysis exploring COVID-19 outcomes within HCWs where this information is available in primary or secondary care medical records, within 24 months prior to the study start date.

HR sub-study

This sub-study will consider only those undertaking active employment in the NHS during the study observation period and will be conducted only in regions where data are available.

Additional ethnicity related predictors include religious belief, nationality, immigration status, and country of birth. Additional occupation predictors include primary area of work, role description, full/part time, patient/non-patient facing [18], time in current role, organisation type, salary grade, length of service, and seniority. Additional exposure related predictors include absence information and total hours worked. Changes in these predictors will be captured throughout the study period. Time varying covariates will be incorporated to enable the assessment of the impact of changes in COVID-19 exposure over time.

Patient and Public Involvement

We have been working with organisations that regulate and represent ethnic minority HCWs, including the GMC, NMC, Royal Colleges, and professional associations such as the British Association of Physicians of Indian Origin. They were involved in identifying research questions and deciding study methodology, and are included as members of the study delivery team or collaborators. These organisations are part of the stakeholder group, meeting monthly to monitor study progress and outputs, provide advice on study delivery, and disseminate research findings. Representatives from ethnic minority healthcare professionals, including those who have contracted COVID-19, will also input into this stakeholder group. A Professional Expert Panel (PEP) of doctors, nurses, and ancillary staff from varying ethnic backgrounds and genders, will meet bi-monthly to provide advice and lived experience related to certain aspects of the project.

For peer review only - http://bmjopen.bmj.com/site/about/guidelines.xhtml

For peer review only

STATISTICAL ANALYSIS PLAN

Statistical principles

Reporting will be in line with STROBE [25] and RECORD [26] guidelines. Any dissemination of study findings will follow best-practice guidelines for deductive disclosure. Only aggregate data will included in publications, 95% confidence intervals will be reported throughout and p-values<0.05 will indicate statistical significance. Multiple testing adjustments will not be made but emphasis will be on effect size rather than statistical significance. Deviations to this protocol and analysis plan will be outlined with justification in the final reporting of the results.

Sample size

Sample size will be driven by the number of HCWs captured by the registration databases, with complete exposure information and successful linkage to healthcare data. The analysis plan is informed by likely event rates for each outcome, undertaking more sophisticated analyses for outcomes with sufficient power to provide meaningful results.

We will consider our largest primary data source (ESR) which captures 1.4 million NHS employees in England and Wales, for a conservative estimate of potential precision. The COVID-19 Infection Survey estimate 6.2% (95% CI 5.0% to 7.6%) of adults in England would be seropositive for anti-SARS-CoV-2 antibodies [27]. Broadly assuming an equal infection rate in Wales, and that HCWs captured by the ESR represent the general population with a similar infection rate, we could expect to capture approximately 86,600 HCWs with history of COVID-19. A large proportion of the infected population will be asymptomatic, and a recent US study projected that 63% of infected cases went undiagnosed [28]. Assuming a similar diagnosis rate in England and Wales, we could expect to identify 32,042 COVID-19 cases. Of adults diagnosed with SARS-CoV-2 infections the estimated hospitalisation rate is 25% [29] and mortality rate is 6.4% [28]. We could therefore expect to observe a minimum of 8,000 hospitalisations and 2,050 deaths resulting from COVID-19.

NHS workforce statistics data show 79.2% of the NHS workforce is White, 10.0% Asian, and 6.1% Black. If associations between ethnicity and COVID-19 outcomes represent those observed in the general population, we could report differences between COVID-19 infection for Asian and Black ethnic categories compared to White with 0.1% and 0.2% precision respectively. Similarly, in those with confirmed SARS-CoV-2 infection we could report differences in COVID-19 mortality between Asian and Black ethnic categories, compared to White, with 0.9% and 0.8% precision respectively.

We will supplement the ESR with other HR databases and professional registration datasets, including SWISS, with an additional 170,000 HCWs in Scotland, and the GMC, with an additional 336,000 GPs not captured by ESR. This increases the total number of HCWs incorporated into our analyses and should ensure sufficient numbers of outcome events.

Missing data

Missing information for ethnicity, occupation, and other predictor variables will be reported and missing data patterns investigated. To improve completeness, we will utilise information from multiple data sources. Those with completely missing ethnicity information will be excluded from the

For peer review only - http://bmjopen.bmj.com/site/about/guidelines.xhtml

main analysis. Comorbidity information will be identified using primary care records; thus, absence of comorbidity codes will be interpreted as absence of comorbidity (i.e. not missing).

We will report the coverage of each analysis and the number (%) of HCWs included. Sensitivity to the exclusion of missing data will be assessed using multiple imputation methods.

Baseline characteristics

The frequency (%) of HCWs by ethnicity and occupational group will be reported. Core predictors (age, sex, comorbidity, and deprivation) will be summarised by ethnicity and occupational group using frequency (%) for categorical measures and mean (standard deviation) or median (interquartile range) for continuous. We will compare core predictors between those with recorded and missing ethnicity information.

For the HR sub-study, additional ethnicity, occupation, and exposure predictors will be summarised alongside the core predictors where available. Changes in roles, exposure, and absences over time will be described across ethnicity category and occupational group.

All baseline data will be presented for the whole cohort and by country.

A flowchart, using the STROBE format, will present cohort selection for both the broad and HR sub-study analyses, with exclusions explained. Average and overall follow-up time will be summarised for each cohort, by ethnicity, occupational group, and country.

Outcome incidence

Adjusted annual and quarterly incidence rates, per 100,000 healthcare workers, for each outcome will be calculated for each country where available. Meta-analysis methods will combine the country-specific estimates to give an overall UK incidence rate estimate. Adjusted incidence rates by ethnicity and occupation will be calculated in the same way. All incidence rates will be adjusted for the four core predictors (age, sex, comorbidities, and deprivation) using standardisation methods. The incidence rates of each outcome will be reported as the rate of cases in the final UK HCW cohort and within subgroups who have experienced preceding COVID-19 events (Table 3 : COVID-19 outcomes and definitions, identified via listed data sources, to be asessed in UK healthcare worker cohort and within listed subgroups, via listed analysis approach in the planned study). The time between diagnosis and outcomes will be summarised and cumulative incidence curves will be plotted.

Associations between ethnicity, occupation, and COVID-19 outcomes

The relationship between ethnicity and COVID-19 outcomes will be assessed using Poisson, logistic, and flexible parametric time-to-event regression models (Table 3 : COVID-19 outcomes and definitions, identified via listed data sources, to be asessed in UK healthcare worker cohort and within listed subgroups, via listed analysis approach in the planned study). For each outcome, multivariable models will incorporate ethnic category and occupational group, and their interactions, adjusted for the four core predictors. The analyses will be conducted by country, and country-specific results combined using meta-analysis methods to produce UK estimates. We will report incidence rate ratios, odds ratios, or hazard ratios with 95% confidence intervals and p-values. Forest plots and cumulative

For peer review only - http://bmjopen.bmj.com/site/about/guidelines.xhtml

incidence curves will be produced. Interactions between ethnicity and other predictors will be presented graphically to aid interpretation. Model assumptions will be assessed.

We will use directed acyclic graphs to map hypothesised causal associations between ethnicity, occupational group, the core predictors, and COVID-19 outcomes. Mediation analysis methods will then be applied to disentangle the direct, indirect, and mediated interactive effects of ethnic category and COVID-19 outcomes. The direct effects estimated using the approach are likely to be related to unmeasured explanatory factors, including experiences of structural discrimination and racism, rather than any genetic predispositions for differing outcomes.

HR sub-study

We will conduct the analysis separately for all countries where both HR and outcomes data are available, country specific results will be presented.

Each outcome will be evaluated using the same regression methods above. Multivariable models will incorporate ethnicity, occupation, and the core predictors as before, whilst extended to incorporate additional predictor information. An ethnicity model will additionally adjust for religion, nationality, and country of birth and incorporate their interactions with ethnicity, if appropriate. An occupation model will additionally adjust for primary area of work, role description, patient/non-patient facing, time in current role, place of work, pay grade, and seniority. Finally, a COVID-19 exposure model will additionally adjust for absence information and total hours worked as time varying exposures, to assess the impact of changes in COVID-19 exposure over time. Forest plots and cumulative incidence curves will be produced. Interactions between ethnicity and other predictors, and the effects of time varying predictors, will be presented graphically to aid interpretation.

Sensitivity and additional analyses

Dependent on the number of HCWs and events captured, we intend to conduct the following additional analyses:

- **Incorporating BMI and smoking into broad analysis**: adjust for BMI and smoking in COVID-19 outcome models if recorded by EHRs either during, or in the 24 months prior to, the study period.
- **Incorporating grouping by organisation:** incorporate clustering of HCWs within organisations by fitting hierarchical models to account for within organisation correlation and between organisation heterogeneity.
- **Granular analysis within the "Asian/Asian British" ethnicity category**: investigate differences in COVID-19 outcomes between HCWs with South Asian (Indian, Pakistani, and Bangladeshi), East Asian (Chinese), and Other Asian backgrounds.
- **Granular analysis within the "medical staff" occupational group:** investigate differences in COVID-19 outcomes between medical and dental subcategories.
- **Subgroup analysis within hospital workers**: investigate COVID-19 outcomes in hospital workers and cluster HCWs within hospitals using hierarchical models.
- **Subgroup analysis of ancillary workers**: investigate COVID-19 outcomes in ancillary workers through trusts that employ them directly.
- **Missing ethnicity:** explore approaches to deal with missing ethnicity information.

For peer review only - http://bmjopen.bmj.com/site/about/guidelines.xhtml

- **Missing data:** use multiple imputation to assess the robustness of our findings to missing data.

For peer review only

For peer review only - http://bmjopen.bmj.com/site/about/guidelines.xhtml

ETHICS AND DISSEMINATION;

Ethical approval for the UK-REACH programme has been obtained via the expedited HRA COVID-19 processes (REC ref: 20/HRA/4718, IRAS ID: 288316). A complimentary investigation into the ethical and legal implications of linking professional registration and healthcare data was commissioned in a parallel work package, the results of which are contained in a published policy report (https://uk-reach.org/main/publications/). In brief, the report considers how the relevant legal framework applies to UK-REACH, discusses the implications of carrying out large-scale data linkage and analysis in a trusted research environment, and highlights the particular ethical issues arising in the context of research on healthcare workers' ethnicity and the COVID-19 pandemic. Ultimately, the report provides a framework for using and linking sensitive data in this project in a way that is ethically, legally, and socially acceptable. The study outlined in this protocol is being guided by the relevant recommendations made within the policy report framework. All research data will be anonymised via the Secure Anonymised Information Linkage (SAIL) Databank before release to researchers. Study results will be submitted for publication in an open access peer-reviewed journals, disseminated through reports to Government, and made available on our dedicated website (https://uk-reach.org/).

For peer review only

For peer review only - http://bmjopen.bmj.com/site/about/guidelines.xhtml

DISCUSSION AND LIMITATIONS

As COVID-19 spreads, the number of cases requiring medical intervention will continue. It is crucial to maintain the healthcare system in the UK and control secondary transmission of COVID-19, and measures to identify and protect the HCWs most vulnerable to infection and adverse outcomes must be implemented. However, current COVID-19 risks in HCWs, particularly within those from historically disadvantaged populations, are poorly understood.

Published evidence of COVID-19 outcomes in HCWs are generally from small studies, in single centre or region settings outside the UK, which have not investigated associations with ethnicity [30]. COVID-19 studies which utilise observational data from non-random sampling, such as hospital admissions or voluntary participation, may be affected by collider bias [31]. One such study assesses outcomes using voluntary self-reported data from symptom trackers only [32], thus results are at risk of selection bias due to reliance on accurate self-reporting of exposures and outcomes. A recent study assessing risks of hospitalisation in HCWs and household members in Scotland [18], had insufficient numbers of HCWs from ethnic minority groups to reliably estimate associations between COVID-19 outcomes and ethnicity. This planned study is, to the authors' knowledge, the first UK-wide study to assess the relationship between ethnicity and COVID-19 outcomes in HCWs. This large study incorporates ambitious linkage of multiple anonymised human resource, professional registration, and NHS datasets providing UK-wide coverage of clinical and non-clinical HCWs. However, the lack of a single UK-wide database capturing all HCWs actively working limits our ability to identify all HCWs across all regions. We aim to identify non-clinical ancillary staff not directly employed by the NHS by incorporating contracting organisations.

The broad analysis will examine associations between ethnicity, occupational group, and multiple important COVID-19 outcomes in HCWs employed in the UK, while adjusting for core predictors. Using readily available EHR data enables the investigation of complex models of public health resulting in scalable, population-based measurements of disease burden [33]. However, we foresee a trade-off between the scale and depth of the data, e.g. while incorporating professional registration databases captures HCWs in non-NHS sectors, we are unable to identify whether they are actively working (not retired, on parental or long-term sick leave). Thus, a sub-study will utilise detailed NHS human resource databases to enable granular analyses of ethnicity, occupation, and COVID-19 exposures in those actively working during the pandemic. Additional hypothesis generating analyses will be conducted where interesting results are found, for example if the study identified a higher mortality incidence in a particular ethnic group, a more detailed analysis investigating differences in disease severity by ethnic group could be planned.

Implications regarding the quality of the EHR data have been considered; multiple data sources will be utilised to limit the impact of missing information for the main exposures (ethnicity and occupation) and outcomes (diagnosis, hospitalisation, death). However, ethnicity information is notorious for being poorly recorded in EHR data and the missing information is unlikely to be missing at random. Even after taking steps to limit the impact of missing information, utilising a complete-case analysis approach may still introduce some sampling bias. Sensitivity analyses are planned to account for predictors expected to have high proportions of non-random missingness, such as ethnicity, BMI, and smoking status. However, this study will be limited by the subset of COVID-19 cases that are diagnosed

For peer review only - http://bmjopen.bmj.com/site/about/guidelines.xhtml

and recorded within the EHR data. Many HCWs with SARS-CoV-2 infections will not be diagnosed, with a US study projecting up to 63% [28]. Indeed, the study will not capture HCWs with mild or asymptomatic infections in settings where tests are restricted to those with symptoms, and results may be vulnerable to collider bias [31]. Incorporating both suspected and confirmed COVID-19 diagnoses is likely to increase the number of infections captured, though it is also likely to reduce the specificity of diagnosis by incorporating false positive cases.

Findings from this study will directly inform evidence-based guidance to protect and address health inequalities in HCWs during the ongoing pandemic.

For peer review only

For peer review only - http://bmjopen.bmj.com/site/about/guidelines.xhtml

REFERENCES

1. WHO, W.H.O., *Coronavirus disease (COVID-19): weekly epidemiological update*. 2020.
2. Chen, N., et al., *Epidemiological and clinical characteristics of 99 cases of 2019 novel coronavirus pneumonia in Wuhan, China: a descriptive study.* The Lancet, 2020. **395**(10223): p. 507-513.
3. Docherty, A.B., et al., *Features of 20 133 UK patients in hospital with covid-19 using the ISARIC WHO Clinical Characterisation Protocol: prospective observational cohort study.* bmj, 2020. **369**.
4. Wynants, L., et al., *Prediction models for diagnosis and prognosis of covid-19 infection: systematic review and critical appraisal.* bmj, 2020. **369**.
5. Yang, J., et al., *Prevalence of comorbidities and its effects in patients infected with SARS-CoV-2: a systematic review and meta-analysis.* International Journal of Infectious Diseases, 2020. **94**: p. 91-95.
6. Patel, A.P., et al., *Race, Socioeconomic Deprivation, and Hospitalization for COVID-19 in English participants of a National Biobank.* medRxiv, 2020.
7. Iacobucci, G., *Covid-19: Deprived areas have the highest death rates in England and Wales*. 2020, British Medical Journal Publishing Group.
8. Aldridge, R.W., et al., *Black, Asian and Minority Ethnic groups in England are at increased risk of death from COVID-19: indirect standardisation of NHS mortality data.* Wellcome Open Research, 2020. **5**(88): p. 88.
9. Harrison, E.M., et al., *Ethnicity and outcomes from COVID-19: the ISARIC CCP-UK prospective observational cohort study of hospitalised patients.* 2020.
10. Mathur, R., et al., *Ethnic differences in COVID-19 infection, hospitalisation, and mortality: an OpenSAFELY analysis of 17 million adults in England.* medRxiv, 2020.
11. Martin, C.A., et al., *Socio-demographic heterogeneity in the prevalence of COVID-19 during lockdown is associated with ethnicity and household size: Results from an observational cohort study.* EClinicalMedicine, 2020: p. 100466.
12. Pan, D., et al., *The impact of ethnicity on clinical outcomes in COVID-19: A systematic review.* EClinicalMedicine, 2020: p. 100404.
13. Chowkwanyun, M. and A.L. Reed Jr, *Racial health disparities and Covid-19—caution and context.* New England Journal of Medicine, 2020.
14. Pareek, M., et al., *Ethnicity and COVID-19: an urgent public health research priority.* The Lancet, 2020. **395**(10234): p. 1421-1422.
15. Lee, C., *"Race" and "ethnicity" in biomedical research: how do scientists construct and explain differences in health?* Social science & medicine, 2009. **68**(6): p. 1183-1190.
16. Khunti, K., et al., *Is ethnicity linked to incidence or outcomes of covid-19?* 2020, British Medical Journal Publishing Group.
17. Koh, D., *Occupational risks for COVID-19 infection.* Occupational medicine (Oxford, England), 2020. **70**(1): p. 3.
18. Shah, A.S., et al., *Risk of hospitalisation with coronavirus disease 2019 in healthcare workers and their households: a nationwide linkage cohort study.* medRxiv, 2020.
19. Cook, T., E. Kursumovic, and S. Lennane, *Exclusive: deaths of NHS staff from covid-19 analysed.* Health Service Journal, 2020. **22**.
20. Levene, L.S., et al., *COVID-19 cumulative mortality rates for frontline healthcare staff in England.* British Journal of General Practice, 2020. **70**(696): p. 327-328.
21. Ford, D.V., et al., *The SAIL Databank: building a national architecture for e-health research and evaluation.* BMC health services research, 2009. **9**(1): p. 157.
22. Drew, D.A., et al., *Rapid implementation of mobile technology for real-time epidemiology of COVID-19.* Science, 2020.

For peer review only

23. Seidu, S., et al., *The impact of obesity on severe disease and mortality in people with SARS-CoV-2: A systematic review and meta-analysis.* Endocrinology, Diabetes & Metabolism, 2020: p. e00176.
24. Williamson, E., et al., *OpenSAFELY: factors associated with COVID-19-related hospital death in the linked electronic health records of 17 million adult NHS patients.* MedRxiv, 2020.
25. Von Elm, E., et al., *The Strengthening the Reporting of Observational Studies in Epidemiology (STROBE) statement: guidelines for reporting observational studies.* Annals of internal medicine, 2007. **147**(8): p. 573-577.
26. Nicholls, S.G., et al., *The reporting of studies conducted using observational routinely-collected health data (RECORD) statement: methods for arriving at consensus and developing reporting guidelines.* PLoS One, 2015. **10**(5): p. e0125620.
27. ONS, O.f.N.S., *Coronavirus (COVID-19) Infection Survey pilot: England, 31 July 2020*. 2020.
28. Gao, R. and K.H. Rosenlof, *Mortality rate and estimate of fraction of undiagnosed COVID-19 cases in the US in March and April 2020.* medRxiv, 2020.
29. Verity, R., et al., *Estimates of the severity of coronavirus disease 2019: a model-based analysis.* The Lancet infectious diseases, 2020.
30. Chou, R., et al., *Epidemiology of and risk factors for coronavirus infection in health care workers: a living rapid review.* Annals of internal medicine, 2020.
31. Griffith, G., et al., *Collider bias undermines our understanding of COVID-19 disease risk and severity.* medRxiv, 2020.
32. Nguyen, L.H., et al., *Risk of COVID-19 among front-line health-care workers and the general community: a prospective cohort study.* The Lancet Public Health, 2020.
33. Hemingway, H., et al., *Big data from electronic health records for early and late translational cardiovascular research: challenges and potential.* European heart journal, 2018. **39**(16): p. 1481-1495.
34. Menni, C., et al., *Real-time tracking of self-reported symptoms to predict potential COVID-19.* Nature medicine, 2020: p. 1-4.

For peer review only

For peer review only - http://bmjopen.bmj.com/site/about/guidelines.xhtml

END MATTER

Authors' contributions

MPareek is corresponding author for this manuscript and attests that all persons listed as authors meet the ICMJE criteria for authorship.

Funding acquisition: CJohn, DFord, KAbrams, KKhunti, LGray, MPareek, MTobin.

Conceptualisation: CMelbourne, COrton, DFord, KAbrams, KKhunti, LGray, LTeece, MPareek.

Writing original draft: CMelbourne, COrton, KAbrams, LGray, LTeece.

Revision and editing: CJohn, CMartin, DFord, DMcAllister, KAbrams, KKhunti, MPareek, MTobin.

Data acquisition, analysis, or interpretation: CMelbourne, COrton, DFord, KAbrams, LGray, LTeece, MPareek.

Funding statement

This publication presents independent research supported by a grant from the MRC-UK Research and Innovation (MR/V027549/1) and the Department of Health and Social Care through the National Institute for Health Research (NIHR) rapid response panel to tackle COVID-19. Core funding was also provided by NIHR Biomedical Research Centres. The views expressed in this publication are those of the authors and not necessarily those of the National Health Service (NHS), the MRC, the NIHR, or the Department of Health and Social Care.

This work is carried out with the support of BREATHE -The Health Data Research Hub for Respiratory Health [MC_PC_19004] in partnership with SAIL Databank. BREATHE is funded through the UK Research and Innovation Industrial Strategy Challenge Fund and delivered through Health Data Research UK.

Competing interests statement

LTeece is supported by the UK National Institute for Health Research (NIHR) Applied Research Collaboration East Midlands (ARC EM).

LGray leads the NIHR ARC EM Data2Health theme.

KKhunti is Director for the University of Leicester Centre for BME Health, Trustee of the South Asian Health Foundation, national NIHR ARC lead for Ethnicity and Diversity, and a member of Independent SAGE. He is supported by the NIHR ARC EM and the NIHR Leicester Biomedical Research Centre.

CJohn holds a Medical Research Council Clinical Research Training Fellowship (MR/P00167X/1).

KAbrams is supported by Health Data Research UK and as a NIHR Senior Investigator Emeritus (NF-SI-0512-10159). He has served as a paid consultant, providing unrelated methodological and strategic advice, to the pharmaceutical and life sciences industry generally and has received unrelated research funding from Association of the British Pharmaceutical Industry, European Federation of Pharmaceutical Industries & Associations, Pfizer, Sanofi and Swiss Precision Diagnostics. He is a Partner and Director of Visible Analytics Limited, a healthcare consultancy company.

For peer review only

MPareek reports grants and personal fees from Gilead Sciences and personal fees from QIAGEN, outside the submitted work.

Data sharing

As the proposed study involves data on unconsented participants, we will be unable to share individual level data once obtained. Study analysis code will be made available following completion of the study and prior to publication of the study manuscript.

Acknowledgements

This study makes use of anonymised data held in the Secure Anonymised Information Linkage (SAIL) Databank. We would like to acknowledge all the data providers who make anonymised data available for research.

This work uses data provided by participants of the COVID-19 Symptoms Study, developed by ZOE Global Limited with scientific and clinical input from King's College London. We would also like to acknowledge all data providers who made anonymised data available for research. We wish to acknowledge the collaborative partnership that enabled acquisition and access to the de-identified data, which led to this output. The collaboration was led by BREATHE, the Health Data Research Hub for Respiratory Health, in partnership with SAIL Databank. We wish to acknowledge the input of ZOE Global Limited and King's College London in their development and sharing of the data, and their input into the understanding and contextualisation of data for COVID-19 research. All research conducted has been completed under the permission and approval of SAIL independent Information Governance Review Panel (IGRP) project number 1120.

The UK-REACH Study Collaborative Group including: Manish Pareek, University of Leicester (Chief Investigator); Laura Gray, University of Leicester; Laura Nellums, University of Nottingham; Anna Guyatt, University of Leicester; Catherine Johns, University of Leicester; Chris McManus, University College London; Katherine Woolf, University College London; Ibrahim Akubakar, University College London; Amit Gupta, Oxford University Hospitals; Keith Abrams, University of York; Martin Tobin, University of Leicester; Louise Wain, University of Leicester; Sue Carr, University Hospital Leicester; Edward Dove, University of Edinburgh; Kamlesh Kunti, University of Leicester; David Ford, University of Swansea; Robert Free, University of Leicester.

For peer review only

TABLES

Table 1: Coverage of human resources and professional registration databases utilised to identifiy UK health care worker cohort for the planned study

Registry	Approximate Size	Country Coverage	Occupational coverage
**Electronic Staff Record (ESR)**	1.4 million	England[1] and Wales	Staff directly employed by NHS England and Wales.
**Scottish Workforce Information Standard System (SWISS)**	163K	Scotland	Staff directly employed by NHS Scotland.
**General Medical Council (GMC)**	336k	England, Wales, Scotland and Northern Ireland	All doctors[2].
**Nursing & Midwifery Council (NMC)**	706k	England, Wales, Scotland and Northern Ireland	Nurses, midwives, student nurses and nursing associates[3].
**General Dental Council (GDC)**	110k	England, Wales, Scotland and Northern Ireland	Dentists, dental care professionals[4] and dental practices.
**General Optical Council (GOC)**	30k	England, Wales, Scotland and Northern Ireland	Optometrists, dispensing opticians, student opticians.
**General Pharmaceutical Council (GPhC)**	94k	England, Wales and Scotland	Pharmacists and pharmacy technicians.
**Pharmaceutical Society Northern Ireland (PSNI)**	3k	Northern Ireland	Pharmacist and trainee pharmacists.
**Health and Care Professions Council (HCPC)**	280k	England, Wales, Scotland, Northern Ireland, and international[5]	Art therapists, biomedical scientists, chiropodists, podiatrists, clinical scientists, dietitians, hearing aid dispensers, occupational therapists, operating department practitioners, orthoptists, paramedics, physiotherapists, practitioner psychologists, prosthetists, orthotists, radiographers, speech and language therapists.

[1]Two foundation trusts in England do not currently use ESR.
[2]Including those in UK Foundation Year 1 and 2 posts, general practitioners, and specialist consultants.
[3]In England only.
[4]Dental care professionals include clinical dental technicians, dental hygienists, dental nursed, dental technicians, dental therapists, orthodontic therapists.
[5]International professionals will not be included in this study as linkage to EHRs, thus assessment of outcomes, will not be possible.

For peer review only

Table 2: ONS ethnicity groupings

Broad ethnic categories	Ethnic groups
**White**	English/Welsh/Scottish/Northern Irish/British
	Irish
	Gypsy or Irish Traveller
	Any other White background
**Mixed/Multiple ethnic groups**	White and Black Caribbean
	White and Black African
	White and Asian
	Any other Mixed/Multiple ethnic background
**Asian/Asian British**	Indian
	Pakistani
	Bangladeshi
	Chinese
	Any other Asian background
**Black/African/Caribbean/Black British**	African
	Caribbean
	Any other Black/African/Caribbean background
**Other ethnic group**	Arab
	Any other ethnic group

For peer review only

Table 3 : COVID-19 outcomes and definitions, identified via listed data sources, to be asessed in UK healthcare worker cohort and within listed subgroups, via listed analysis approach in the planned study

Outcomes		Definitions	Data sources	Subgroups	Analysis approach (output)
**COVID-19 diagnosis**	Confirmed COVID-19 diagnosis	A positive swab test for SARS-CoV-2 during the study period	PHE Pillar 1 (PHE labs or NHS hospital) and Pillar 2 (wider population) testing data[1]		Poisson regression (IRR)
	Suspected COVID-19 diagnosis	Self-reporting of either a positive SARS-CoV-2 test or combination of symptoms shown to be predictive of positive testing[2] during the study period or suspected case SNOMED CT codes (NHS Digital) recorded during the study period	COVID-19 Symptom Study app and primary care data		Poisson regression (IRR)
**Hospitalisation**	All hospitalisations	Any hospital admission recorded during the study period	Secondary care and CHESS data	Confirmed COVID-19 diagnosis	Logistic regression (OR)
	COVID-19 hospitalisations	hospital admissions with COVID-19 specific ICD-10 codes[3] during the study period or positive swab test for SARS-CoV-2 recorded during or within 28 days prior to admission	Secondary care, CHESS, PHE Pillar 1 (PHE labs or NHS hospital) and Pillar 2 (wider population) testing data[1]	Confirmed COVID-19 diagnosis	Logistic regression (OR)
	COVID-19 ITU admissions	Admissions to ITU during the study period	Secondary care, CHESS, and intensive care audit data	Confirmed COVID-19 diagnosis	Logistic regression (OR)
**Mortality**	All-cause mortality	Any death registration recorded during the study period	ONS mortality, primary care, and secondary care data	Confirmed COVID-19 diagnosis<br>COVID-19 hospitalisations<br>Covid-19 ITU admissions	Flexible parametric time-to-event regression (HR)
	Mortality due to COVID-19	any death registration with COVID-19 specific ICD-10 codes[3] recorded for the primary or secondary cause of death during the study period	ONS mortality, primary care, and secondary care data	Confirmed COVID-19 diagnosis<br>COVID-19 hospitalisations<br>Covid-19 ITU admissions	Flexible parametric time-to-event regression (HR)

For peer review only - http://bmjopen.bmj.com/site/about/guidelines.xhtml

COVID-19, Coronavirus disease 2019; SARS-CoV-2, severe acute respiratory syndrome coronavirus 2; PHE, Public Health England; NHS, National Health Service; IRR, incidence rate ratio; SNOMED CT, Systematized Nomenclature of Medicine - Clinical Terms; CHESS, COVID-19 Hospitalisation in England Surveillance System; OR, odds ratio; ICD-10, International Classification of Diseases – 10th revision; ITU, Intensive Therapy Unit; ONS, Office for National Statistics; HR, hazard ratio.

[1]PHE Pillar 3 (serology testing for antibodies) and Pillar 4 (blood and swab testing) data will be incorporated if obtained.

[2]Symptoms include loss of smell or taste, fatigue, persistent cough, and loss of appetite [34].

[3]COVID-19 specific ICD-10 codes are U07.1: "COVID-19, virus identified" and U07.2: "COVID-19, virus not identified".

For peer review only

Table 4 : Definitions for core predictors associated with risk of adverse COVID-19 outcomes

Predictor	Definition
**Age**	A continuous measure of age (in years) at the index date (01-02-2020), calculated using date of birth as recorded in HR or professional registration databases.
**Sex**	A categorical measure (female/male/other) as recorded in HR or professional registration databases.
**Comorbidities**	A categorical measure counting the number of comorbidities for each individual (0, 1, or 2 or more) obtained from primary care records and supplemented with secondary care records in analyses conducted in the hospitalised cohort. Comorbidity count will incorporate conditions from the NHS Digital shielded patient list algorithm and others found to be associated with adverse COVID-19 outcomes.
**Socio-economic deprivation**	An ordinal measure using quintiles of the Index of Multiple Deprivation (IMD), the official measure of relative deprivation for small areas in each country, based on residential postcode as recorded in HR or professional registration databases. Where residential postcode is not provided workplace postcode will be used as a surrogate.

For peer review only

FIGURES

The above diagram and style was interpreted from an initial data flow diagram created and provided by Andy Boyd at the University of Bristol. It has been repurposed and amended to illustrate data flows specific to the UK-REACH project by Chris Orton at Swansea University.

Figure 1 : Data flow and linkage of UK-REACH data sources

For peer review only

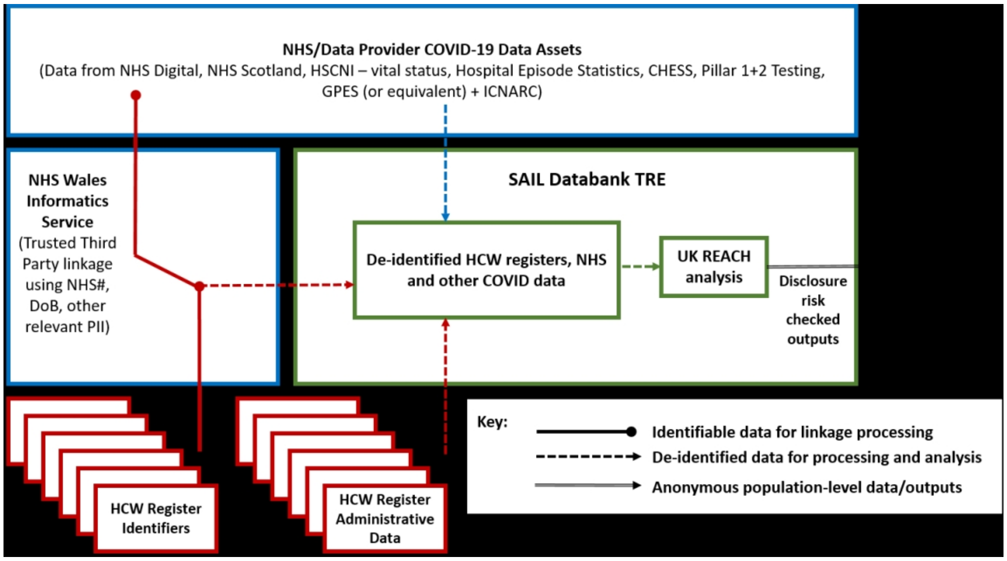

Data flow and linkage of UK-REACH data sources.
The above diagram and style was interpreted from an initial data flow diagram created and provided by Andy Boyd at the University of Bristol. It has been repurposed and amended to illustrate data flows specific to the UK-REACH project by Chris Orton at Swansea University.

The RECORD statement – checklist of items, extended from the STROBE statement, that should be reported in observational studies using routinely collected health data.

	Item No.	STROBE items	Location in manuscript where items are reported	RECORD items	Location in manuscript where items are reported
**Title and abstract**					
	1	(a) Indicate the study's design with a commonly used term in the title or the abstract (b) Provide in the abstract an informative and balanced summary of what was done and what was found	Title – P1<br>Abstract – P2	RECORD 1.1: The type of data used should be specified in the title or abstract. When possible, the name of the databases used should be included.<br><br>RECORD 1.2: If applicable, the geographic region and timeframe within which the study took place should be reported in the title or abstract.<br><br>RECORD 1.3: If linkage between databases was conducted for the study, this should be clearly stated in the title or abstract.	Title – P1<br>Abstract – P2<br><br>Abstract – P2<br><br>Title – P1
**Introduction**					
Background rationale	2	Explain the scientific background and rationale for the investigation being reported	Introduction – P4		
Objectives	3	State specific objectives, including any prespecified hypotheses	Objectives – P5		
**Methods**					
Study Design	4	Present key elements of study design early in the paper	Study design – P6		
Setting	5	Describe the setting, locations, and relevant dates, including periods of recruitment, exposure, follow-up, and data collection	Data sources – P6-7<br>Observation period – P7		

For peer review only

Participants	6	*(a) Cohort study* - Give the eligibility criteria, and the sources and methods of selection of participants. Describe methods of follow-up<br>*Case-control study* - Give the eligibility criteria, and the sources and methods of case ascertainment and control selection. Give the rationale for the choice of cases and controls<br>*Cross-sectional study* - Give the eligibility criteria, and the sources and methods of selection of participants<br><br>*(b) Cohort study* - For matched studies, give matching criteria and number of exposed and unexposed<br>*Case-control study* - For matched studies, give matching criteria and the number of controls per case	Study population – P7<br><br>Observational period – P7<br><br>NA	RECORD 6.1: The methods of study population selection (such as codes or algorithms used to identify subjects) should be listed in detail. If this is not possible, an explanation should be provided.<br><br>RECORD 6.2: Any validation studies of the codes or algorithms used to select the population should be referenced. If validation was conducted for this study and not published elsewhere, detailed methods and results should be provided.<br><br>RECORD 6.3: If the study involved linkage of databases, consider use of a flow diagram or other graphical display to demonstrate the data linkage process, including the number of individuals with linked data at each stage.	Study population – P7<br><br>NA<br><br>Figure1
Variables	7	Clearly define all outcomes, exposures, predictors, potential confounders, and effect modifiers. Give diagnostic criteria, if applicable.	Main exposures – P8<br>Outcomes – P8 & Table 3<br>Predictors – P8 & Table 4	RECORD 7.1: A complete list of codes and algorithms used to classify exposures, outcomes, confounders, and effect modifiers should be provided. If these cannot be reported, an explanation should be provided.	Main exposures – P8<br>Outcomes – P8 & Table 3<br>Predictors – P8 & Table 4
Data sources/ measurement	8	For each variable of interest, give sources of data and details of methods of assessment (measurement).<br>Describe comparability of assessment methods if there is more than one group	Data sources – P6-7<br>Main exposures – P8<br>Outcomes – P8 & Table 3<br>Predictors – P8 & Table 4		

For peer review only

Bias	9	Describe any efforts to address potential sources of bias	Discussion – P13		
Study size	10	Explain how the study size was arrived at	Sample size – P10		
Quantitative variables	11	Explain how quantitative variables were handled in the analyses. If applicable, describe which groupings were chosen, and why	Main exposures – P8<br>Predictors – P8 & Table 4		
Statistical methods	12	(a) Describe all statistical methods, including those used to control for confounding<br>(b) Describe any methods used to examine subgroups and interactions<br>(c) Explain how missing data were addressed<br>(d) *Cohort study* - If applicable, explain how loss to follow-up was addressed<br>*Case-control study* - If applicable, explain how matching of cases and controls was addressed<br>*Cross-sectional study* - If applicable, describe analytical methods taking account of sampling strategy<br>(e) Describe any sensitivity analyses	Statistical analysis plan – P10-12<br><br>Sensitivity and Additional analyses – P12<br>Missing data – P10-11<br>HR sub-study – P12<br><br>Sensitivity and Additional analyses – P12		
Data access and cleaning methods		..		RECORD 12.1: Authors should describe the extent to which the investigators had access to the database population used to create the study population.	Data linkage – P7 & Figure 1

For peer review only

				RECORD 12.2: Authors should provide information on the data cleaning methods used in the study.	N/A - protocol
Linkage		..		RECORD 12.3: State whether the study included person-level, institutional-level, or other data linkage across two or more databases. The methods of linkage and methods of linkage quality evaluation should be provided.	Data linkage – P7
**Results**					
Participants	13	(a) Report the numbers of individuals at each stage of the study (*e.g.*, numbers potentially eligible, examined for eligibility, confirmed eligible, included in the study, completing follow-up, and analysed)<br>(b) Give reasons for non-participation at each stage.<br>(c) Consider use of a flow diagram	N/A - protocol<br>N/A - protocol<br>N/A - protocol	RECORD 13.1: Describe in detail the selection of the persons included in the study (*i.e.,* study population selection) including filtering based on data quality, data availability and linkage. The selection of included persons can be described in the text and/or by means of the study flow diagram.	N/A - protocol
Descriptive data	14	(a) Give characteristics of study participants (*e.g.*, demographic, clinical, social) and information on exposures and potential confounders<br>(b) Indicate the number of participants with missing data for each variable of interest<br>(c) *Cohort study* - summarise follow-up time (*e.g.*, average and total amount)	N/A - protocol<br>N/A - protocol<br>N/A - protocol		
Outcome data	15	*Cohort study* - Report numbers of outcome events or summary measures over time<br>*Case-control study* - Report numbers in each exposure	N/A - protocol		

For peer review only

		category, or summary measures of exposure *Cross-sectional study* - Report numbers of outcome events or summary measures			
Main results	16	(a) Give unadjusted estimates and, if applicable, confounder-adjusted estimates and their precision (e.g., 95% confidence interval). Make clear which confounders were adjusted for and why they were included<br>(b) Report category boundaries when continuous variables were categorized<br>(c) If relevant, consider translating estimates of relative risk into absolute risk for a meaningful time period	N/A - protocol<br>N/A - protocol<br>N/A - protocol		
Other analyses	17	Report other analyses done—e.g., analyses of subgroups and interactions, and sensitivity analyses	N/A - protocol		
**Discussion**					
Key results	18	Summarise key results with reference to study objectives	N/A - protocol		
Limitations	19	Discuss limitations of the study, taking into account sources of potential bias or imprecision. Discuss both direction and magnitude of any potential bias	Discussion and limitations -P12-13	RECORD 19.1: Discuss the implications of using data that were not created or collected to answer the specific research question(s). Include discussion of misclassification bias, unmeasured confounding, missing data, and changing eligibility over time, as they pertain to the study being reported.	Discussions and limitation - P12
Interpretation	20	Give a cautious overall interpretation of results considering objectives,	N/A - protocol		

		limitations, multiplicity of analyses, results from similar studies, and other relevant evidence			
Generalisability	21	Discuss the generalisability (external validity) of the study results	Discussion and limitations -P12-13		
**Other Information**					
Funding	22	Give the source of funding and the role of the funders for the present study and, if applicable, for the original study on which the present article is based	Funding statement P14		
Accessibility of protocol, raw data, and programming code		..		RECORD 22.1: Authors should provide information on how to access any supplemental information such as the study protocol, raw data, or programming code.	Data sharing – P14

*Reference: Benchimol EI, Smeeth L, Guttmann A, Harron K, Moher D, Petersen I, Sørensen HT, von Elm E, Langan SM, the RECORD Working Committee. The REporting of studies Conducted using Observational Routinely-collected health Data (RECORD) Statement. *PLoS Medicine* 2015; in press.

*Checklist is protected under Creative Commons Attribution (CC BY) license.

For peer review only
